# Unravelling the physiological and anatomical basis of divergent adaptations in cultivated and wild tomatoes

**DOI:** 10.1093/jxb/eraf390

**Published:** 2025-09-04

**Authors:** Showkat A Ganie, Guillaume Forget, Joana Amaral, Shellie A Wall, Pallavi Singh, Johannes Kromdijk, Elizabete Carmo-Silva, Tracy Lawson

**Affiliations:** School of Life Sciences, University of Essex, Colchester CO4 3SQ, UK; INRAE, UMR BIOGECO, University of Bordeaux, Pessac 33615, France; Lancaster Environment Centre, Lancaster University, Lancaster LA1 4YQ, UK; Department of Plant Biology & Institute for Genomic Biology, School of Intergrative Biology, College of Liberal Arts and Sciences, 1402 Inst for Genomic Biology, University of Illinois at Urbana-Champaign, 1206 W Gregory Dr., Urbana, IL 61801, USA; School of Life Sciences, University of Essex, Colchester CO4 3SQ, UK; Department of Plant Sciences, University of Cambridge Cambridge CB2 3EA, UK; Lancaster Environment Centre, Lancaster University, Lancaster LA1 4YQ, UK; School of Life Sciences, University of Essex, Colchester CO4 3SQ, UK; Department of Plant Biology & Institute for Genomic Biology, School of Intergrative Biology, College of Liberal Arts and Sciences, 1402 Inst for Genomic Biology, University of Illinois at Urbana-Champaign, 1206 W Gregory Dr., Urbana, IL 61801, USA; University of Nottingham, UK

**Keywords:** Crop productivity, grafting, natural variation, photosynthesis, stomata, tomatoes

## Abstract

Distinct physiological and anatomical traits can lead to substantial variation in photosynthetic efficiency among plant varieties, which may, in turn, impact agronomically important traits. We conducted a comprehensive comparative analysis of leaf physiology, anatomy, and biochemistry in *Solanum lycopersicum* (LEA), a modern inbred variety suited for the processing industry, and *Solanum pennellii* (Lost, accession LA5240), a drought-tolerant, green-fruited wild species, to investigate differences in photosynthetic performance and stomatal physiology. Lost exhibited higher photosynthetic capacity due to both biochemical and anatomical features. Chlorophyll fluorescence revealed that photosynthesis operates at a higher rate in Lost, due to greater electron sink capacity and efficient electron flow through the photosystems. Lost also showed higher Rubisco content as well as greater chlorophyll *a*/*b* ratio and total soluble protein levels than LEA, demonstrating investments in carbon capture relative to light harvesting to support superior photosynthetic performance at higher light intensities. Equal stomatal numbers on the abaxial and adaxial surface for Lost supported its greater leaf thickness and higher photosynthetic capacity, whilst LEA’s greater stomatal density on the abaxial surface is typical of commercial broadleaf crops. Grafting experiments demonstrated that LEA scions grafted onto Lost rootstocks displayed improved photosynthesis compared with non-grafted LEA and LEA self-grafted plants, demonstrating successfully transferred enhanced photosynthetic traits from rootstock of Lost to LEA scions. Our study highlights the photosynthetic advantages of Lost and suggests avenues for enhancing tomato productivity through trait transfer.

## Teaser

The wild tomato *Solanum pennellii* has specialized leaf design and photosynthetic machinery that boost photosynthetic capacity, which can be transferred to commercial tomato plants through non-GM grafting techniques.

## Introduction

Global food security faces challenges from population growth and climate change, with the population projected to reach nearly 10 billion by 2050 (United Nations, 2017). Climate change causes environmental stresses like heat stress and water scarcity, reducing crop yields (Sutanto *et al.*, 2024). Tomatoes are a key part of the food chain in many areas of the world as they are rich in essential nutrients and antioxidants, which have been linked to reduced cancer and heart disease risks (Collins *et al.*, 2022). Over 180 million metric tons are produced annually in diverse climates (FAO, 2021), whilst their short growing cycle (90–120 d) makes them adaptable to changing climates (FAO, 2015). However, climate change threatens tomato production with temperature fluctuations, water scarcity, and extreme weather, which would significantly reduce yields and shift optimal growing periods (Bhandari *et al.*, 2021; Onyeneke *et al.*, 2023). These challenges highlight the urgent need to develop tomato varieties and cultivation methods that maintain high productivity under stress. Our study directly addresses this by examining photosynthetic performance, stomatal characteristics, and leaf structure in cultivated and wild tomatoes to determine physiological impacts on improved resilience and growth. Greater photosynthesis has been linked to higher yields as well as improved stress resilience (López-Calcagno *et al.*, 2020; Miglani *et al.*, 2021; Paul, 2021; Wang *et al.*, 2021; Raines, 2022; Walter and Kromdijk, 2022; Croce *et al.*, 2024; Keller *et al.*, 2024; Khan *et al.*, 2024). Photosynthesis is the fundamental process in plants that transforms light energy into chemical energy, producing sugars that serve as the building blocks for plant growth and metabolism (Erb and Zarzycki, 2018). There are two stages to photosynthesis: the light reactions (or electron transport chain), which convert absorbed light energy into chemical energy producing ATP and the reductant NADPH, and the carbon reactions in which these are used within the Calvin–Benson–Bassham cycle to assimilate CO_2_. Manipulations in both electron transport and CO_2_ assimilation have been shown to be closely linked to crop performance and serve as potential targets for enhanced production (Fryer *et al.*, 1998; López-Calcagno *et al.*, 2020; Wang *et al.*, 2021; Raines, 2022; Walter and Kromdijk, 2022).

For photosynthesis to take place, CO_2_ must enter the leaf through stomata, microscopic pores found on the epidermis of leaves and stems (as well as other green plant parts) (Simkin *et al.*, 2019; Lawson and Milliken, 2023). Regulation of pore aperture plays a critical role in facilitating gaseous exchange between the leaf interior and the surrounding atmosphere (Lawson and Vialet-Chabrand, 2019). Guard cells surrounding these pores determine the opening and closing of stomata in response to environmental conditions including light intensity, humidity, and atmospheric CO_2_ concentration (Lawson, 2009) as well as internal cues (Lawson and Blatt, 2014). However, stomatal opening also leads to water loss through transpiration, which is important for nutrient uptake and evaporative cooling but can become limiting under reduced water availability. Stomatal conductance is a measure of the maximum rate of gaseous exchange assessed as H_2_O efflux from the leaf and is positively correlated with photosynthetic rates (Battle *et al.*, 2024) but negatively correlated with water use efficiency (Lawson and Blatt, 2014; Buckley, 2017; Lemonnier and Lawson, 2024), reflecting the fundamental trade-off between maximizing CO_2_ uptake for photosynthesis and minimizing water loss (Franks and Farquhar, 2007).

Stomatal conductance and behaviour are determined by anatomical features such as stomatal density (SD) as well as behaviour characteristics including pore opening and rapidity of responses. Studies have shown that higher SD can enhance stomatal conductance, typically leading to increased CO_2_ assimilation and greater photosynthetic rates under fluctuating light conditions (Sakoda *et al.*, 2020); however, this can come at the expense of increased water loss and lower water use efficiency (Lawson and Blatt, 2014). The relationship between SD and yield is complex, because plants with lower SD generally use less water and can therefore maintain or even improve yields under conditions of water stress (Caine *et al.*, 2019). Given this complexity, developing crops with optimized stomatal characteristics has become a major focus in crop improvement programs.

Domestication of crops including tomato has led to a limited genetic bottleneck, reducing the diversity within cultivated varieties. However, natural variation within landraces and wild relatives offers new avenues for crop improvement and resilience by optimizing photosynthetic and stomatal traits (Bai and Lindhout, 2007; Faralli *et al.*, 2024). Tomato (*Solanum lycopersicum*) has 12 wild relative species with significant genetic diversity and therefore potential for improving specific traits for agronomic gains (Yoshiyama *et al.*, 2024) including photosynthesis. In fact, several wild tomato species (*Solanum* spp.) like *S. pimpinellifolium* (wild cherry tomato) and *S. chilense* have been shown to have greater photosynthetic rates, stress tolerance, and water-use efficiency than commercial varieties (Venema *et al.*, 1999; Haque *et al.*, 2015; Bigot *et al.*, 2023; Yoshiyama *et al.*, 2024). *Solanum chmielewskii*, *S. habrochaites*, and *S. pennellii* (accession LS2355) exhibit faster photosynthetic induction and stomatal conductance than cultivated tomato varieties under fluctuating light conditions (Yoshiyama *et al.*, 2024). *Solanum pennellii* (LS2355) also exhibits smaller sized and denser stomata compared with cultivated varieties, offering an intriguing potential source of adaptive variation (Yoshiyama *et al.*, 2024). Likewise, *S*. *habrochaites* and *S*. *peruvianum* show higher SD and Rubisco activity than cultivated varieties (Zeist *et al.*, 2018; Zhou *et al.*, 2018). The photosynthetic net CO_2_ assimilation rates in the high-altitude wild species *S. arcanum* LA385 are greater than those of the cultivar Moneymaker at sub-optimal temperatures (16/14 °C) (Dinh *et al.*, 2019). Based on these differences in photosynthetic attributes, novel alleles from wild species could be leveraged to boost the photosynthetic performance of cultivated tomatoes, especially under resource-limited or stressful conditions.

Exploiting natural variation and techniques such as grafting, a traditional horticultural technique commonly used in commercial practices, stands out as a promising non-GM strategy for enhancing performance and quality of different crops, including tomatoes. There is evidence indicating that grafting cultivated tomato varieties onto rootstocks of wild species can significantly improve resistance to various abiotic stresses, such as salinity, drought, and extreme temperatures (Kumar *et al.*, 2017; Zhang *et al.*, 2019; Singh *et al.*, 2020; Alves *et al.*, 2021; Fuentes-Merlos *et al.*, 2022; Bahadur *et al.*, 2024; Bahadur and Kumar, 2024). Given the high potential of wild species to improve photosynthesis in cultivated tomato, we studied the physiological basis of high photosynthetic efficiency in the ‘Lost’ (LA5240) accession of wild, green-fruited, drought tolerant *S. pennellii*. Lost is a wild tomato species native to the arid regions of South America. While this accession has remained largely unexplored until recently, it has demonstrated considerable potential for improving cultivated tomato varieties (Torgeman *et al.*, 2024). A modern determinate tomato inbred line. ‘LEA’, specifically bred for open-field cultivation (Rochsar *et al.*, 2025), was also included for comparative study purposes. Comparing different species highlights traits that confer stress tolerance, which might not be evident were only one species to be studied.

To address the urgent need for climate-resilient and high-yielding tomato varieties, this study leverages the contrasting adaptations of cultivated tomato (LEA) and a drought-tolerant wild species (Lost). We tested the hypothesis that the wild tomato Lost exhibits superior photosynthetic traits compared with the cultivated LEA and asked if this was due to underlying differences in anatomy and/or biochemistry. Finally, we explored the potential of grafting Lost rootstocks to LEA scions as a mechanism to transfer beneficial physiological traits (such as enhanced photosynthetic performance or altered stomatal patterning) to the cultivated variety. By systematically exploring these questions, our work aims to identify key physiological and anatomical traits that could be harnessed to improve tomato resilience and productivity under climate stress.

## Materials and methods

### Plant growth

The seeds of two tomato species, *Solanum lycopersicum* (LEA) and *S. pennellii* (Lost), were sown in plastic trays filled with compost and allowed to germinate in a growth cabinet (Reftech BV, Sassenheim, Netherlands) at the University of Essex. The conditions were set to approximately 200 µmol m⁻² s⁻¹ of photosynthetically active radiation (PAR), a 14 h light–10 h dark photoperiod, an average temperature of around 15 °C, and about 60% relative humidity (RH). After about 2 weeks when seeds had germinated, the seedlings were transplanted into 1.5 litre pots (15 cm diameter, 12 cm deep) containing F2S compost and transferred to a temperature-controlled glasshouse. The plants were grown (with six biological replicates for each line) under greenhouse conditions of temperature 24 °C, 65% RH, ambient CO_2_, and 400 µmol m^−2^ s^−1^ light. About 3–4 weeks after transplanting, plants were used for photosynthetic and other physiological measurements.

### Measurement of plant height and whole plant leaf area

About 2 weeks after transplantation, the whole plant leaf area and height of LEA and Lost were measured every other day for a duration of 36 d using a PlantEye F600 multispectral 3D scanner (Phenospex). This system non-destructively quantifies the true leaf surface area by accounting for leaf curvature and overlap within the intact plant canopy, providing a more ecologically relevant measure than traditional 2D destructive methods.

### Gas exchange and chlorophyll fluorescence

All gas exchange and chlorophyll fluorescence measurements were performed on the first fully developed leaf (from the top). An LI-6800 (LI-COR, Lincoln, NE, USA) was used to carry out the gas exchange measurements. Environmental settings were as follows: flow 500 µmol s^−1^, RH 65%, CO_2_ (for step change and light response) 400 µmol mol^−1^, fan speed 10 000 rpm, temperature (Txchg) 24 °C, and light depending on the program used. For chlorophyll fluorescence, the same settings for humidity, CO_2_, and temperature as for gas exchange measurements were used.

A Dual-KLAS (kinetic LED array spectrophotometer) near-infrared (NIR) spectrophotometer (Heinz Walz, Effeltrich, Germany) was used for specific analysis of chlorophyll fluorescence and in-depth analysis of electron transport. The Dual-KLAS NIR spectrophotometer was integrated with a Walz CFS3000 gas exchange chamber, allowing measurements under controlled environmental conditions: 65% RH, 400 µmol mol^−1^ CO_2_, and a temperature of 24 °C. This instrument employs four differential NIR signals to non-invasively assess the redox states of P700 [photosystem I (PSI)], plastocyanin, and ferredoxin, along with chlorophyll fluorescence parameters, following the manufacturer’s protocols. Quantum yields of PSI and photosystem II (PSII), along with redox states of P700, plastocyanin, and ferredoxin, were analysed as described by Schreiber (2017).

For light response curves in both gas exchange and deep phenotyping analyses, a CO_2_ level of 400 µmol mol^−1^ and different light levels of 1800, 1500, 1300, 1100, 900, 700, 550, 400, 250, 150, 100, and 50 µmol m^−2^ s^−1^ were used. For CO_2_ response, a light level of 1500 µmol m^−2^ s^−1^ was used and plants were subjected to different CO_2_ levels of 400, 250, 150, 100, 50, 400, 550, 700, 900, 1100, 1300, and 1500 µmol mol^−1^. For step changes, plants were first dark-adapted at 100 µmol m^−2^ s^−1^ light and then exposed to 1000 µmol m^−2^ s^−1^ in four steps of 25 min each.

### Stomatal density

SD in LEA and Lost was assessed by taking leaf surface impressions from both the abaxial and adaxial sides using silicone impression material (Xantopren, Heraeus, Hanau, Germany), following the method outlined by Weyers and Johansen (1985). Impressions were made at the same site on the leaf after gas exchange measurements were completed. SD was quantified using light microscopy (Olympus BX60, Olympus UK & Ireland, Southend-on-Sea, UK) at ×50 magnification. For each impression, an average of three technical replicates was analysed. Stomatal pore length was measured along the longitudinal axis of a stoma, from the pore’s apex to its base, with five stomata measured per leaf and six biological replicates per genotype.

### Simultaneous independent measurements of gas exchange from abaxial and adaxial leaf surfaces

Gas exchange was measured independently and simultaneously from both the abaxial and adaxial leaf surfaces using a custom-built ‘split chamber’ gas exchange system, following the protocol of Wall *et al.* (2022). This system allows for separate quantification of net photosynthetic assimilation (*A*) and stomatal conductance (*g*_s_) from each leaf surface, providing detailed insight into surface-specific gas exchange dynamics. Environmental conditions during measurements matched those used for the LI-6800 system (RH 65%, CO_2_ 400 µmol mol^−1^, temperature 24 °C).

### Leaf thickness and leaf mass per area

Leaf thickness was measured using hand-held MultispeQ v2.0 (PhotosynQ, East Lansing, MI, USA) instrument. For both tomato species, one fully expanded leaf from the uppermost part of the canopy from six biological replicates was measured. To determine the leaf mass per area (LMA), three leaf discs (total area 1.65 cm^2^) from a top (fully expanded) leaf were dried at 65 °C for 24 h in an oven. The dry weight was measured, and LMA determined by dividing leaf dry mass by leaf area. Six biological replicates were used for each line.

### Microtomy

To study the leaf anatomy of LEA and Lost, rectangular leaf sections were cut out of the leaves and fixed by vacuum infiltrating 5% glutaraldehyde aqueous solution in glass universals. The fixed leaf sections were treated with 1× phosphate buffered saline (30 min), washed with MiliQ water (30 min) and rinsed with serial dilutions of 50%, 70%, 90% and 100% ethanol (30 min each). Leaf sections were then left in 100% ethanol overnight at room temperature. The leaf sections were treated with 100% ethanol and freshly prepared LR White acrylic resin mixture (1:1) for 1 h. Leaf sections were then treated for 2–3 h with 100% LR White resin with intermittent shaking. The excess resin was then decanted and the leaf sections placed inside the capsules filled with fresh resin. The capsules were kept at 60 °C inside an oven for 24 h to allow polymerization of resin. The leaf section-containing polymerized resin capsules were appropriately sliced using an Ultracut microtome (Reichert Jung) and the resin slices were mildly heated on glass slides, stained with toluidine blue, and observed under a light microscope. From these stained sections, qualitative differences in mesophyll structure and density between LEA and Lost were visually assessed under the light microscope, providing anatomical context for observed physiological differences. No quantitative measurements were taken from these sections.

### Quantification of light absorbance of leaves

Light absorption was measured from four leaves of LEA and Lost using an integrating sphere and broadband absorbance with a PAR sensor. The amount of light reflected (reflectance) and transmitted (transmittance) by the leaf was used to calculate the absorbance as Absorbance=1−(Reflectance+Transmittance), which was used in the calculation of electron transport rate (ETR).

### Quantification of Rubisco activity and content and chlorophyll content

The first fully developed leaf at the top of the canopy, corresponding to that used for gas exchange measurements, was sampled, from which three leaf discs (total area 1.65 cm^2^) were collected, avoiding any shading (six replicates for each line). The collected leaf discs were rapidly frozen in liquid nitrogen and stored at −80 °C for further analyses. Rubisco initial activity was determined per leaf area. Rubisco amount and carboxylation activity in leaf extracts were determined radiometrically as described in Ashton *et al.* (2024). Rubisco activity was measured by incorporation of [^14^C]CO_2_ into 3-phosphoglycerate at 30 °C. Initial activities in leaf extracts were measured immediately after protein extraction, while total activities were assayed after incubation of leaf extract for 3 min in the assay mix without ribulose-1,5-bisphosphate (RuBP); preliminary tests comparing 1–7 min incubation times showed that 3 min allows full carbamylation of Rubisco catalytic sites. Rubisco activation state was calculated as the ratio of initial to total activity. The abundance of Rubisco in leaf extracts was determined by incubation with excess of Rubisco’s synthetic inhibitor [^14^C]carboxyarabinitol-1,5-bisphosphate (^14^C-CABP). Separation of unbound ^14^C-CABP from the Rubisco–^14^C-CABP complex using a Sephadex column followed by detection of Rubisco–^14^C-CABP complex radioactivity allowed Rubisco quantification. Total soluble proteins (TSP) in the leaf extracts were quantified using the Bradford method (Bradford, 1976). Chlorophyll content in leaf extracts was determined by measuring absorbance of an ethanolic extract (20 μl leaf protein extract: 480 μl of 95% ethanol) at 649 nm and 665 nm (Wintermans and de Mots, 1965).

### Grafting

The grafting of LEA and Lost plants was performed approximately 4 weeks after transplantation. Four types of grafts were created: LEA scions on LEA rootstocks (LEA/LEA), Lost scions on Lost rootstocks (Lost/Lost), LEA scions on Lost rootstocks (LEA/Lost), and Lost scions on LEA rootstocks (Lost/LEA). Non-grafted plants served as controls. The rootstock and scion were cut at matching angles (whip grafting), and the cuts were interlocked and secured with grafting clips. The grafted plants were grown in the greenhouse under the previously described conditions. Gas exchange and SD measurements on the scion leaves were performed 30 d after grafting, once the graft junctions had successfully established, to determine any changes in photosynthetic potential and stomatal behaviour resulting from different rootstock–scion combinations.

For all gas exchange measurements in the grafted plants, we selected leaves of similar developmental stage and position as those used in the earlier experiments. This approach ensured comparability between grafted and non-grafted treatments, with all measurements performed under the same environmental conditions and using consistent protocols.

### Stomatal kinetics estimation

To describe the temporal response of *g*_s_, an analytical model derived from that used by McAusland *et al.* (2016) was used (Equation 1). The model described the temporal response of *g*_s_ (stomatal opening) using a time constant (τ, min), an initial time lag (λ, min), and a steady-state *g*_s_ (*g*_s,max_, mmol m^−2^ s^−1^) reached at high photosynthetic photon flux density (PPFD) using the following equation:


(1) $$g_{s}=(g_{s,\max}-g_{s,\min})e^{e^{\left(\frac{\lambda -t}{\tau }+1\right)}}+g_{s,\min}$$

where *t* is the time, where time 0 is the point at which PPFD was increased from 100 to 1000 µmol m^−2^ s^−1^, and *g*_s,min_ (mmol m^−2^ s^−1^) is the initial value of stomatal conductance (before the change in PPFD). In this equation, the time constant τ is a measure of the rapidity of response of *g*_s_ independent of the amplitude of variation in *g*_s_.

Parameters τ and λ were estimated using the NLS (nonlinear least-squares) function with ‘port’ algorithm from the ‘stats’ package.

A second parameter combining rapidity and amplitude of the response, the maximum slope (Sl_max_), was used to describe the maximal slope of the *g*_s_ response to the step-change in PPFD:


(2) $$Sl_{\max}=\frac{\tau (g_{s,\max}-g_{s,\min})}{e}$$

We also determined the time to reach 95% of *A*_max_.

### Statistical analysis

All statistical analyses were performed using R v.4.2.2 (R Core Team, 2022). The data were analysed using Shapiro’s test for normality and Levene’s test for homogeneity of variance. Variables that met these assumptions were analysed using Student’s *t*-test. If homogeneity of variance was not met, Welch’s test was used. Variables that did not meet normality were analysed using the Mann–Whitney test. To compare more than two groups, ANOVA (post-hoc Tukey’s test) was used after checking assumptions; otherwise, the Kruskal–Wallis test (post-hoc Dunn’s test) was performed. A significance level of *P*<0.05 was considered for all tests. From the CO_2_ response curve analysis, the photosynthetic parameters including maximum rate of carboxylation (*V*_c,max_), maximum electron transport rate (*J*_max_), maximum assimilation rate (*A*_max_), and the triose phosphate utilization rate (TPU) were determined using the Farquhar–Berry–von Caemmerer model implemented via the ‘plantecophys’ package (Duursma, 2015). *A–Q* curves were parameterized to determine the light saturation of photosynthesis (*A*_sat_) (https://cran.r-project.org/web/packages/photosynthesis/vignettes/light-response.html) based on the model by Marshall and Biscoe (1980).

## Results

### Comparative growth patterns of LEA and Lost: leaf area, height, and implications for adaptation to drought

**Fig. 1.**
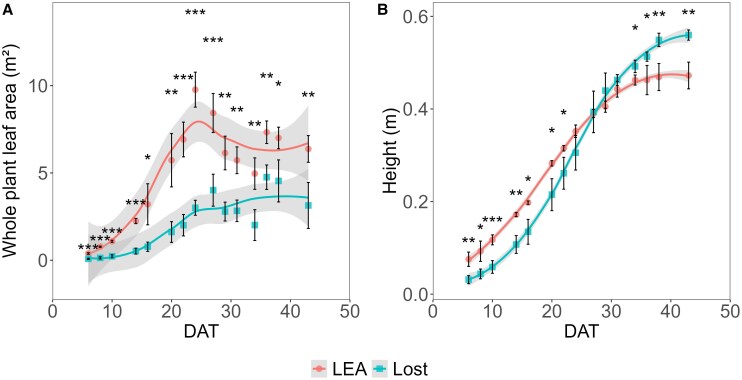
Whole plant leaf area and plant height of LEA and Lost. The growth patterns of LEA and Lost, particularly in terms of whole plant leaf area and height, were quantified throughout the experiment. (A) LEA consistently exhibited a significantly higher whole plant leaf area than Lost (B) Although LEA initially had greater plant height, Lost began to surpass LEA in height around 29 d after transplanting (DAT). Data are presented as mean values ±standard deviation (*n*=3–6). Statistical differences were assessed using Student's *t*-test: *P<0.05, **P<0.01, ***P<0.001.

In order to quantify the varied patterns of growth for LEA and Lost, whole plant leaf area and height were quantified over time. LEA had consistently higher (*P*<0.05) leaf area compared with Lost over the course of development (Fig. 1A), owing to larger and more leaves per branch. While LEA initially was taller (*P*<0.05) than Lost, the latter started to overtake LEA in height ca. 29 d after transplanting (DAT), reaching 0.55 m by 40 DAT, whilst LEA only achieved 0.44 m (Fig. 1B).

### Photosynthetic capacity

#### *A*–CO_2_ analysis

**Fig. 2.**
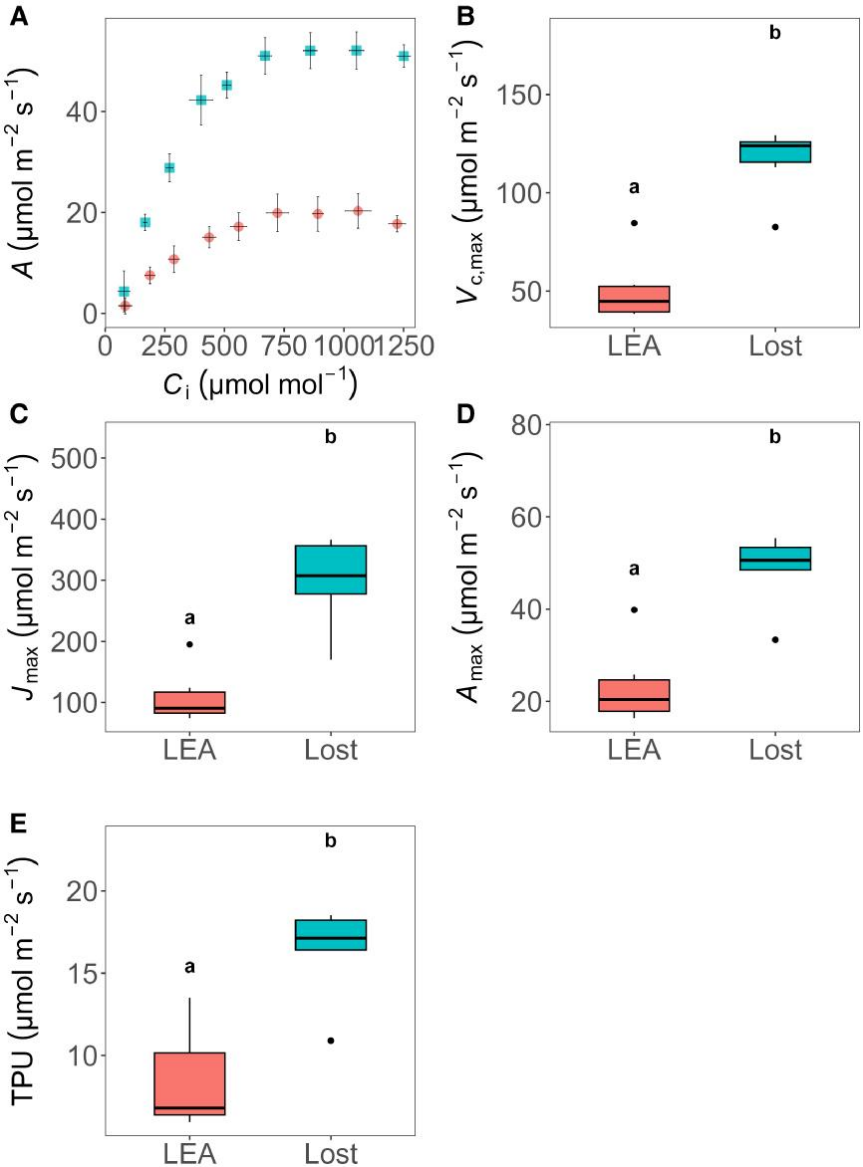
CO_2_ response curve and deduced parameters for photosynthetic assimilation. (A) The net photosynthetic assimilation (*A*) was significantly higher in Lost compared with LEA under different CO_2_ levels. The CO_2_ response curve analysis revealed significant differences in key photosynthetic parameters between LEA and Lost. (B–E) Maximum rate of carboxylation (*V*_c,max_) (B), maximum electron transport rate (*J*_max_) (C), maximum assimilation rate (*A*_max_) (D), and triose phosphate utilization rate (TPU) (E) were all significantly higher in Lost compared with LEA (*P*<0.01, *P*<0.001, *P*<0.001, and *P*<0.05, respectively). Statistical differences were assessed using Student's *t*-test for (C, D) and the Mann–Whitney test for (B, E). Different letters denote significant differences between genotypes (*P*<0.05). Data are presented as mean values ±standard deviation (*n*=3–6).

To identify differences in the CO_2_ assimilation rates between LEA and Lost, the rate of photosynthesis (*A*) in response to changes in internal CO_2_ concentration (*C*_i_) was assessed. Our analysis revealed a significantly higher net CO_2_ assimilation in Lost compared with LEA across a range of CO_2_ concentrations (Fig. 2A). With increasing *C*_i_, Lost exhibited a higher photosynthetic rate compared with LEA. Photosynthetic rate in Lost increased rapidly with *C*_i_ up to approximately 500 ppm, reaching a maximum *A* of ca. 50 µmol m^−2^ s^−1^. In contrast, LEA showed a lower *A* across all *C*_i_ concentrations, with *A* saturating at a maximum of around 20 µmol m^−2^ s^−1^. The differences in *A* between Lost and LEA were most pronounced at *C*_i_ concentrations above 300 ppm. The key photosynthetic parameters maximum rate of carboxylation (*V*_c,max_), maximum electron transport rate (*J*_max_), maximum assimilation rate (*A*_max_), and the triose phosphate utilization rate (TPU) were determined from the *A*–*C*_i_ curves. All these parameters were significantly higher (*P<*0.01, *P<*0.001, *P*<0.001, and *P*<0.05, respectively) in Lost compared with LEA (Fig. 2B–E), indicating superior biochemical capacity per unit leaf area in Lost (Fig. 2).

### Deep phenotyping of photosynthetic electron transport and quantum yield variations

**Fig. 3.**
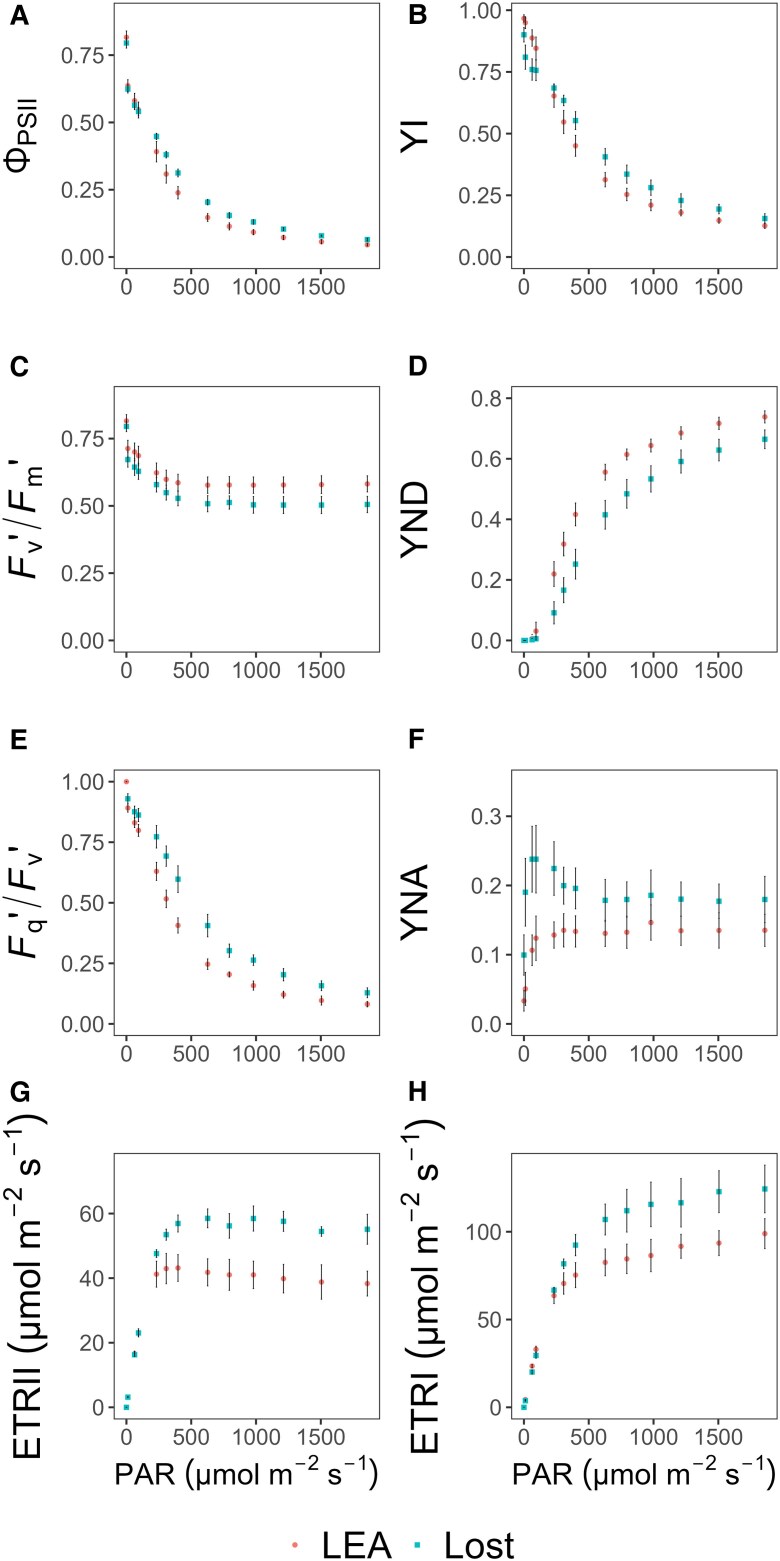
Response of photosystem II and I quantum yields and electron transport rates to increasing light in LEA and Lost. (A, B) Both quantum yield of photosystem II (Φ_PSII_) and quantum yield of photosystem I (YI) decline with increasing photosynthetically active radiation (PAR), with Lost exhibiting higher values at higher PAR levels. (C, E) Chlorophyll fluorescence components, where *F*_v_′/*F*_m_′ (maximum quantum efficiency in the light) remains relatively stable and similar for both genotypes, while *F*_q_′/*F*_v_′ (photochemical factor) decreases with increasing light, with more pronounced decrease in LEA than Lost. (D) photosystem I donor side limitation (YND) increases with light with LEA exhibiting higher values than Lost. (F) Fraction of P700 unable to accept electrons (YNA) shows no significant differences between the two genotypes, except at low light levels where Lost is greater than LEA. (G, H) Relative electron transport rates (ETRII and ETRI), showing greater rates in Lost, particularly at light intensities above 250 µmol m^−2^ s^−1^, while ETRII in LEA declines at PAR above 500 µmol m^−2^ s^−1^. Data are presented as mean values ±standard deviation (*n*=3–6).

Quantum yield of photosystem II (Φ_PSII_) and I (YI) were determined using a Dual/KLAS-NIR spectrophotometer, and as expected, both genotypes exhibited a decline in Φ_PSII_ and YI with increasing PAR (Fig. 3A, B). At PAR levels below 250 µmol m^−2^ s^−1^, no differences between LEA and Lost were observed; however, at higher PAR, both Φ_PSII_ and YI were greater in Lost than LEA. Breaking the operating efficiency of PSII (Φ_PSII_) into the chlorophyll fluorescence components *F*_q_′/*F*_v_′ (the photochemical factor) and *F*_v_′/*F*_m_′ (the maximum quantum efficiency in the light), it was clear that changes in *F*_q_′/*F*_v_′, and therefore electron transport components downstream of PSII, were responsible for the majority of light driven changes in Φ_PSII_ and the differences between LEA and Lost (Fig. 3C, D). To further examine electron flow downstream of the photosystems, we measured PSI donor side limitation (YND; which provides an indication of the fraction of P700 oxidized due to slow or limited electron donation) and acceptor side limitation (YNA; which measures the fraction of P700 unable to accept electrons due to a downstream process limiting electron flow). YND was higher in LEA than Lost at all light levels, whilst no differences in YNA were observed, except at relatively low light levels when Lost was greater than LEA (Fig. 3E, F), which supports the observations that the greater Φ_PSII_ in Lost is due to greater downstream capacity for electrons. The higher relative electron transport rates (ETRs) at both PSII (ETRII) and PSI (ETRI) in Lost at light intensities above 250 µmol m^−2^ s^−1^ (Fig. 3G, H) again support these observations.

**Fig. 4.**
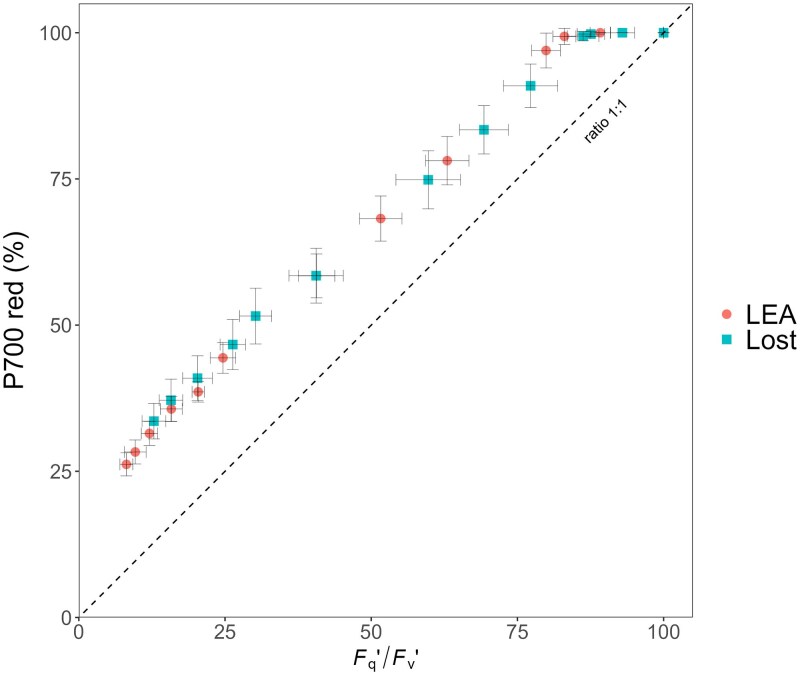
Relationship between the percentage of reduced P700 (P700_red_ %) and photochemical quenching at photosystem II (*F*_q_′/*F*_v_′) in LEA and Lost. The linear relationship indicates a balance of activity between the two photosystems. The dashed line indicates a 1:1 ratio. Data are presented as mean values ±standard deviation (*n*=3–6).

The redox states of key components of the electron transport chain including plastocyanin (PC), PS I reaction centre (P700), and ferredoxin (Fd) as a function of light revealed potential limitations to photosynthetic electron transport under specific conditions. No differences in the redox state of P700 (P700red) between Lost and LEA (Supplementary Fig. S1A) were observed. At low PAR, both LEA and Lost exhibited a highly reduced P700 state (approximately 98–100%). In contrast, the percentage of PC reduction (PC_red_) in LEA was significantly lower at lower light levels and 100% reduction was observed at a lower light intensity than Lost (Supplementary Fig. S1B). The higher PC_red_ at low light levels in Lost indicates that plastocyanin was maintained in a reduced state, most likely from high electron supply from PSII. For ferredoxin reduction state (Fd_red_), LEA showed high variability at low light, ranging from 25% to 45%; in contrast, Lost showed consistently low Fd_red_, which remained constant at all light intensities (Supplementary Fig. S1C). These results seem to indicate that Lost can achieve higher PSI efficiency due to almost fully oxidizing ferredoxin. The strong linear relationship observed between reduced P700 and photochemical quenching at PSII (*F*_q_′/*F*_v_′) in both genotypes (Fig. 4) indicates balance of activity between the two photosystems and further strengthens Calvin cycle and sink activity as the main driver behind photosynthetic differences between the two cultivars.

### Stomatal density and size on the abaxial and adaxial leaf surfaces

**Fig. 5.**
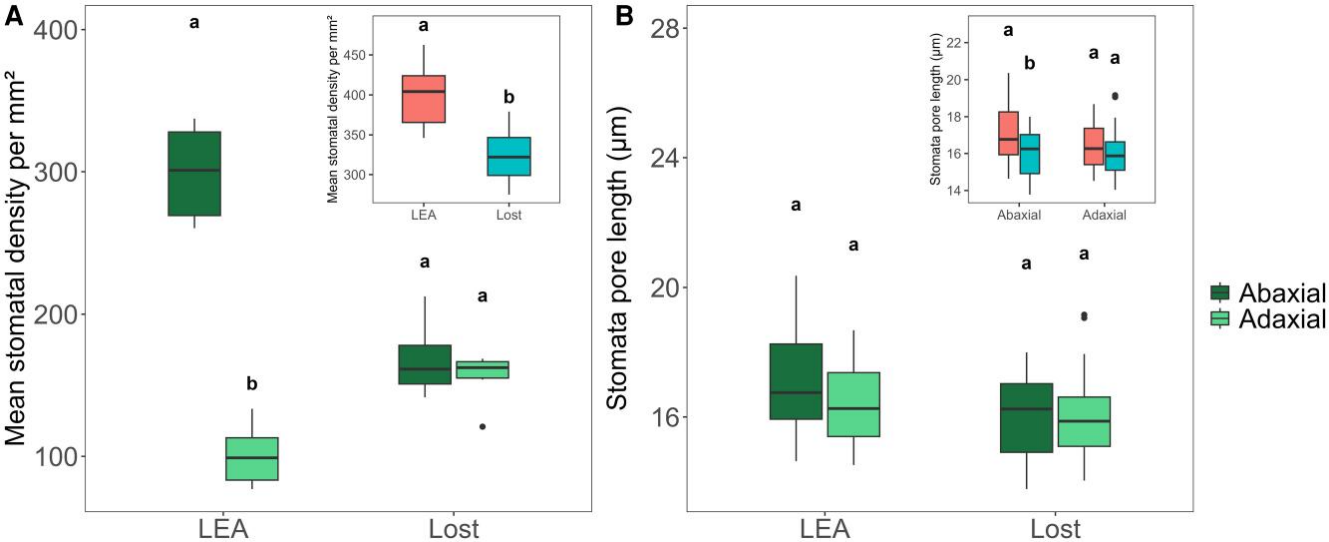
Stomatal density (SD) and pore size on abaxial and adaxial leaf surfaces in LEA and Lost. (A) LEA shows a higher stomatal density on the abaxial leaf surface compared with the adaxial surface (*P*<0.001). In contrast, Lost exhibits no significant difference in SD between the two surfaces. Overall, LEA shows higher SD than Lost (inset) (*P*<0.01). (B) Within each genotype, there was no significant difference between abaxial and adaxial stomatal pore lengths. Comparison of pore length between genotypes for each surface revealed a significant reduction in abaxial pore length in Lost compared with LEA (*P*<0.01), while adaxial pore lengths did not differ significantly (inset). Statistical differences were assessed using Student's *t*-test except when assumptions are not validated, where the Mann–Whitney test was used. Different letters denote significant differences (*P*<0.05) between genotypes. Data are presented as mean values ±standard deviation (*n*=3–6).

To investigate the differences in stomatal density (SD), leaf impressions were taken from both the abaxial and adaxial leaf surfaces. LEA had a significantly higher SD (*P*<0.001) on the abaxial than adaxial surface, while no difference in SD from two surfaces was observed in Lost (Fig. 5A). Moreover, SD on the abaxial leaf surface of LEA was significantly higher compared with the abaxial surface of Lost (*P*<0.001). Conversely, the adaxial surface of LEA showed significantly lower stomatal density than the adaxial surface of Lost (*P*<0.01). Overall, the SD in LEA was significantly higher than Lost (*P*<0.01). No significant differences in pore length were observed between abaxial and adaxial surfaces for the two genotypes (LEA: abaxial 17.2 μm, adaxial 16.8 μm; Lost: abaxial 16 μm, adaxial 15.8 μm). However, a significant difference between genotypes on the abaxial surface was observed: LEA had significantly larger abaxial stomatal pores (17.2 μm) compared with Lost (15.8 μm; *P*<0.05), while adaxial pore lengths were similar between genotypes (Fig. 5B).

### Surface-specific differences in stomatal conductance and net photosynthetic assimilation

**Fig. 6.**
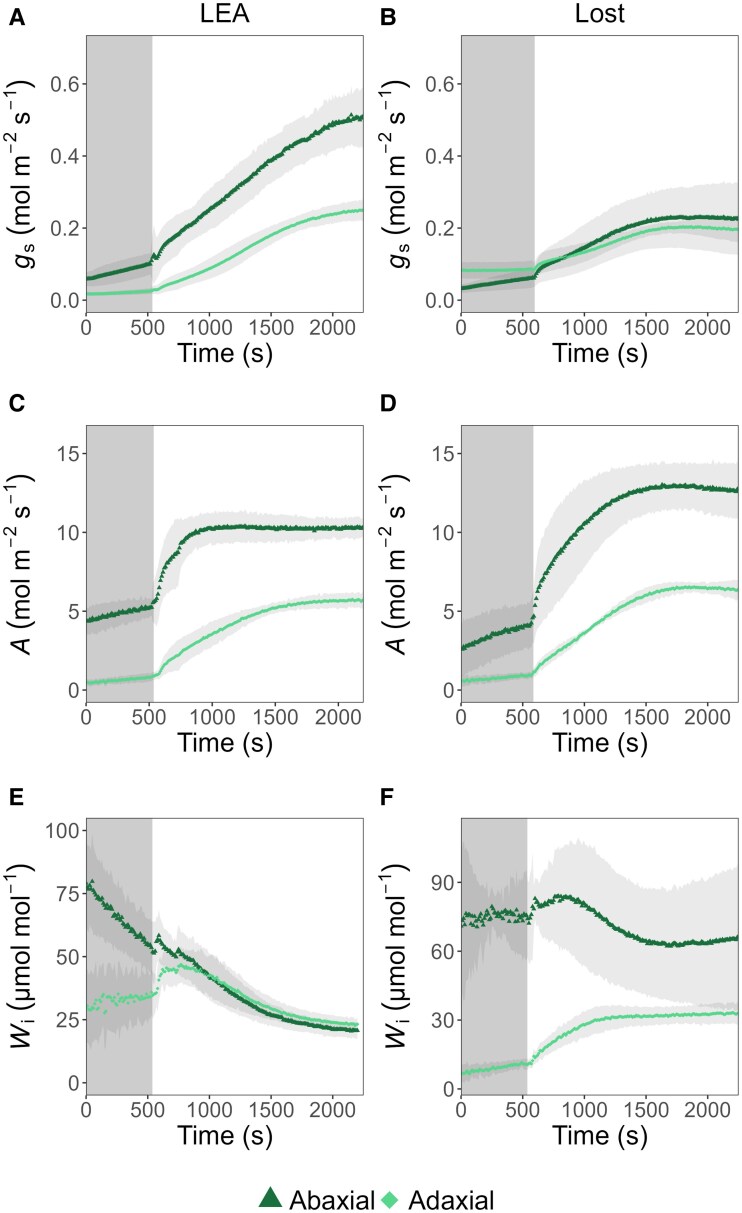
Stomatal conductance (*g*_s_), photosynthetic assimilation (*A*) and intrinsic water use efficiency (*W*_i_) on abaxial and adaxial leaf surfaces in LEA and Lost. (A) *g*_s_ was higher on the abaxial surface than the adaxial surface in LEA. (B) No significant difference in *g*_s_ was observed between the two surfaces in Lost. Overall, LEA exhibited higher *g*_s_ than Lost. (C, D) *A* differed between surfaces in both LEA and Lost, with the abaxial surface performing better than the adaxial surface. Overall, Lost showed higher photosynthesis than LEA. (E, F) *W*_i_ was consistently and substantially higher for the abaxial surface in Lost compared with LEA, while no significant differences were observed between genotypes on the adaxial surface. Data are presented as mean values ±standard deviation (*n*=3–6). Grey rectangular regions represent periods of light transition from 100 to 1000 µmol m^−2^ s^−1^.

To determine if differences between abaxial and adaxial SD and size in LEA and Lost influence *g*_s_ and change photosynthetic physiology, gas exchange measurements from each surface were analysed independently and simultaneously using a novel split chamber (Wall *et al.*, 2022). The response of *g*_s_ and *A* to a step change in light intensity from 100 to 1000 µmol m^−2^ s^−1^ was assessed. Before the change in light intensity, both LEA and Lost exhibited low *g*_s_ values (Fig. 6A, B). After increasing PAR, *g*_s_ increased in both genotypes, with distinct responses observed between the abaxial and adaxial leaf surfaces. In LEA, the abaxial surface showed a higher final *g*_s_ value, reaching approximately 0.5 mol m^−2^ s^−1^, while the adaxial surface reached approximately 0.25 mol m^−2^ s^−1^. In Lost, the difference between abaxial and adaxial surfaces was less pronounced, with the abaxial *g*_s_ reaching approximately 0.25 mol m^−2^ s^−1^ and the adaxial *g*_s_ reaching approximately 0.2 mol m^−2^ s^−1^. Overall, LEA had greater *g*_s_ on both surfaces than Lost. *A*, as expected, also increased after the increase in light intensity in both genotypes. However, surprisingly, *A* in Lost, on the abaxial surface was considerably higher than the adaxial surface and not correlated with *g*_s_, while in LEA, *A* was in agreement with SD and *g*_s_ from the two surfaces (Fig. 6C, D). LEA exhibited a final *A* of approximately 10 µmol m^−2^ s^−1^ for the abaxial surface and approximately 6 µmol m^−2^ s^−1^ for the adaxial surface. In contrast, Lost achieved a higher final *A* of approximately 13 µmol m^−2^ s^−1^ for the abaxial surface and approximately 7 µmol m^−2^ s^−1^ for the adaxial surface.

The response time of *g*_s_ was lower in Lost than LEA, indicting more rapid *g*_s_ kinetics in Lost (Supplementary Fig. S2A), although no differences in the lag time was apparent between genotypes (Supplementary Fig. S2B), and the maximum slope of the response (Sl_max_) was lower (Supplementary Fig. S2C) highlighting the lower magnitude of change. However, the differences in rapidity did not alter the time to reach 95% *A*_max_. Although no differences in the speeds of the *g*_s_ responses were observed between the two leaf surfaces in Lost (Supplementary Fig. S3A), τ and Sl_max_ on the adaxial surface of LEA were significantly lower than the abaxial surface (Supplementary Fig. S3C), resulting in a faster time to reach 95% *A*_max_ (Supplementary Fig. S3D). Intrinsic water use efficiency (*W*_i_), determined from the kinetics responses, was consistently higher on the abaxial surface in Lost compared with LEA, whilst there were no differences between the two cultivars for the adaxial surface (Fig. 6E, F).

### Anatomical features: leaf thickness, leaf mass per area, and mesophyll structure

To determine whether the observed differences in photosynthetic assimilation between LEA and Lost (and the two surfaces) were linked to differences in leaf morphology, thickness and leaf mass per area (LMA)—a proxy for weight of a leaf relative to its size—were compared between the two species (Supplementary Fig. S4A, B). LEA exhibited significantly lower leaf thickness (*P*<0.05), measuring approximately 0.23 mm, compared with 0.32 mm in Lost. Likewise, Lost had an LMA of approximately 49 mg mm^−2^, while LEA exhibited a significantly lower LMA of approximately 34 mg mm^−2^ (*P*<0.05). The observed differences in leaf thickness and LMA suggest variations in leaf structure and resource allocation between the two genotypes. To investigate the differences at the anatomical level between the leaf surfaces of LEA and Lost, we examined leaf cross sections for differences in mesophyll structure and arrangement. Microscopic examination showed that Lost possesses larger and highly packed palisade mesophyll cells as well as higher density of spongy mesophyll cell than LEA (Supplementary Fig. S4C, D).

### Leaf biochemical composition suggests a distinct photosynthetic biochemistry

To investigate if the maximum rate of CO_2_ assimilation of LEA and Lost (Fig. 2A) were relatable to the Rubisco, the activity and content of Rubisco along with the contents of chlorophyll (both chlorophyll *a* and *b*) and total soluble protein (TSP) were determined (Supplementary Fig. S5). Lost showed a mean Rubisco initial activity (*V*_i_) of approximately 15 µmol m^−2^ s^−1^, while LEA exhibited a significantly lower *V*_i_ of approximately 5 µmol m^−2^ s^−1^ (*P*<0.05). In line with the trends observed for *V*_i_, Lost also displayed a significantly higher Rubisco total activity (*V*_t_), with a mean of approximately 30 µmol m^−2^ s^−1^ in comparison with approximately 7 µmol m^−2^ s^−1^ in LEA (*P*<0.05). LEA exhibited a significantly higher Rubisco activation state, with a mean of approximately 80%, while Lost showed a significantly lower activation state of approximately 60% (*P*<0.01) (Supplementary Fig. S5A–C). Rubisco content was approximately five times higher in Lost than LEA (*P*<0.05), which also translated into Lost possessing a significantly higher TSP content of approximately 3 g m^−2^ as compared with 1 g m^−2^ in LEA (*P*<0.05) (Supplementary Fig. S5D, E).

Although there were no significant differences in chlorophyll *a* and chlorophyll *b* content per unit area between Lost and LEA, there was a significant difference in chlorophyll *a*/*b* ratio. LEA exhibited a chlorophyll *a*/*b* ratio of approximately 2, while Lost showed a significantly higher chlorophyll *a*/*b* ratio of approximately 2.2 (*P*<0.05) (Supplementary Fig. S5G–I). Rubisco/total chlorophyll ratio was approximately 0.9 in LEA, while Lost showed a significantly higher ratio of approximately 1.8 (*P*<0.01). Similarly, Lost had a higher TSP/chlorophyll ratio than LEA (*P*<0.05) (Supplementary Fig. S5J, K). Altogether, these results further highlight significant biochemical differences between LEA and Lost leaves, with Lost demonstrating higher Rubisco activity and content, as well as ratios relative to TSP and total chlorophyll compared with LEA.

### Grafting for trait-transfer from Lost to LEA

**Fig. 7.**
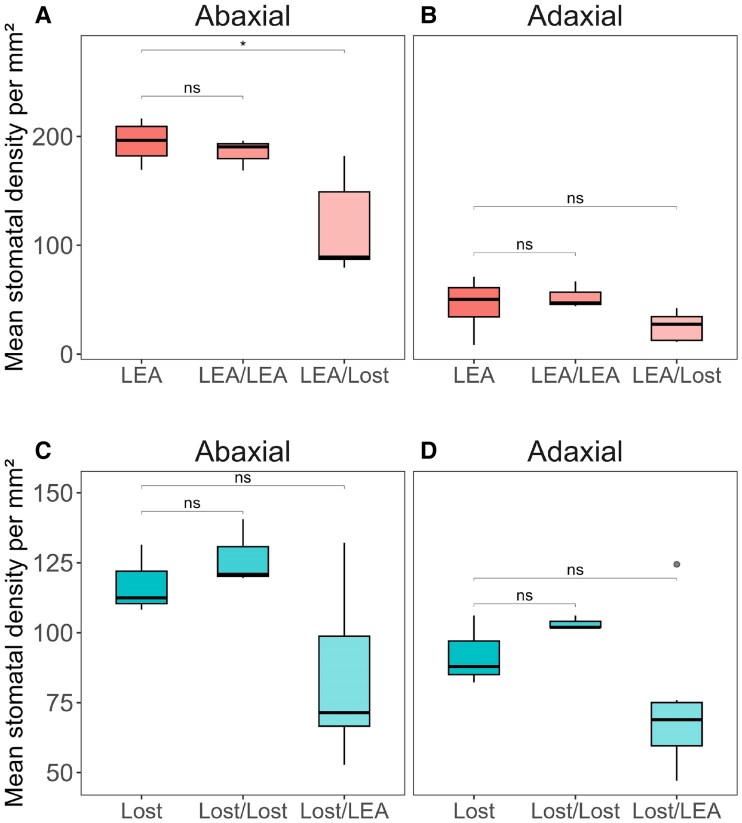
Stomatal density (SD) analysis on abaxial and adaxial leaf surfaces of non-grafted (LEA, Lost), self-grafted (LEA/LEA, Lost/Lost), and reciprocally grafted (lost/LEA, LEA/Lost) plants. (A) On the abaxial side, the stomatal density of LEA/Lost is significantly lower than that of reference LEA. (B) On the adaxial side, SD does not differ between the grafting combinations with respect to non-grafted reference LEA. The difference in SD between the abaxial and adaxial leaf surfaces in LEA grafted onto Lost (LEA/Lost) is reduced compared with non-grafted LEA. (C, D) On the abaxial (C) and adaxial (D) sides, the SD does not differ between the grafting combinations and the non-grafted reference Lost. Grey circle represents an outlier. Data are presented as mean values ±standard deviation (*n*=3–6). Statistical differences were assessed using Student's *t*-test (*P*<0.05), except for self-graphed Lost/Lost where a Mann–Whitney test was used. ‘ns’, no significant difference; *P<0.05.

**Fig. 8.**
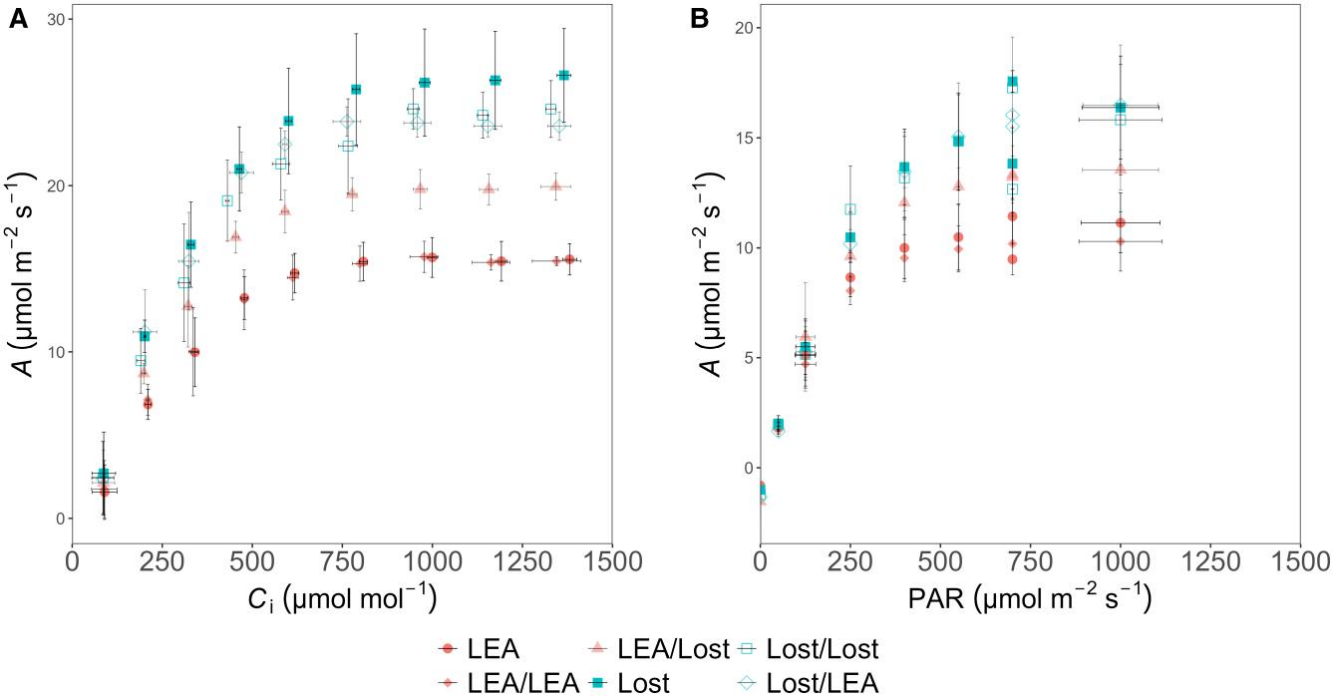
Comparison of photosynthetic performance between LEA and Lost grafting combinations. (A) LEA scions grafted onto Lost rootstocks (LEA/Lost) exhibit enhanced photosynthetic performance compared with non-grafted LEA and LEA self-grafts (LEA/LEA) under various CO_2_ levels. (B) Lost, either non-grafted (Lost), or autografted (Lost/Lost), or grafted on LEA (Lost/LEA), demonstrates higher photosynthesis than LEA (either non-grafted, or autografted, or grafted on Lost). Data are presented as mean values ±standard deviation (*n*=3–6).

In order to determine if the differences in physiological properties described above were driven by shoot or root traits, we employed grafting techniques using the Lost accession as rootstock, while maintaining non-grafted LEA plants as controls. Additionally, we autografted LEA scions onto their own rootstocks. The same grafting combinations were also applied to the Lost accession. To assess whether grafting influenced SD, we analysed SD in grafted LEA and Lost plants (Fig. 7). As expected, SD was generally similar across the abaxial and adaxial leaf surfaces in the Lost, Lost/Lost, and Lost/LEA graft combinations (Fig. 7). In contrast, SD was significantly higher on the abaxial than adaxial surface of LEA, LEA/LEA, and LEA/Lost combinations (*P*<0.001, *P*<0.001, and *P*<0.01, respectively). An interesting observation was that the difference in SD between the abaxial and adaxial leaf surfaces in LEA grafted onto Lost (LEA/Lost) was reduced compared with LEA/LEA and non-grafted LEA. The SD on the abaxial leaf surface of LEA/Lost plants was significantly reduced with respect to non-grafted LEA (*P*<0.05). This suggests that the influence of Lost rootstock may result in a more uniform SD across both leaf surfaces in LEA scions. To follow up these results, we investigated if the different SD patterns also influenced the photosynthesis in the grafted plants (Fig. 8). No differences in *V*_c,max_ or *J*_max_ (Supplementary Fig. S6A, B) were observed in any of the grafted plants. However, as shown previously, *A*_max_ was lower in non-grafted LEA, plateauing around 15–17 µmol m^−2^ s^−1^ in CO_2_ response (Fig. 8A, Supplementary Fig. S6C), whilst the light saturated rate of *A* (*A*_sat_) was 10–12 µmol m^−2^ s^−1^ in light response (Supplementary Fig. S6E), compared with non-grafted Lost, which reaches *A*_max_ of approximately 25–27 µmol m^−2^ s^−1^ and *A*_sat_ of 17–19 µmol m^−2^ s^−1^ in light response (Fig. 8B). Self-grafting in LEA (LEA/LEA) showed no differences in *A* compared with non-grafted controls, whereas LEA scions grafted onto Lost rootstock (LEA/Lost) exhibit significantly enhanced *A*_max_ of approximately 20–22 µmol m^−2^ s^−1^ and higher *A*_sat_ of ca. 15–17 µmol m^−2^ s^−1^ (Fig. 8E). No difference in *A*_sat_ or *A*_max_ were observed between grafted and non-grafted Lost/LEA. These results suggest that rootstock identity significantly influences scion photosynthetic performance and that grafting offers a promising way to enhance photosynthetic traits from wild relatives to cultivated tomato lines without genetic modification.

## Discussion

Tomato (*Solanum lycopersicum*) is a crop of global importance, but productivity is increasingly threatened by climate change-induced drought stress. This study presents a comprehensive comparative analysis of leaf physiology and anatomy between cultivated tomato (*S. lycopersicum*, LEA) and its wild relative, *S. pennellii* (Lost, accession LA5240), which is renowned for its exceptional drought tolerance (Torgeman *et al.*, 2024). By examining various aspects of leaf structure, stomatal characteristics, and photosynthetic performance, we aimed to uncover the underlying mechanisms that contribute to the superior photosynthetic efficiency and adaptation of Lost to water-limited environments. Our findings reveal a suite of coordinated physiological and anatomical adaptations in Lost that collectively enhance photosynthetic performance. Moreover, grafting LEA onto Lost rootstocks resulted in improved photosynthetic performance, highlighting the potential importance of roots and associated root–shoot signalling or hydraulic architecture on above-ground processes. The insights gained from this study provide valuable targets for crop improvement programs aimed at developing more photosynthetically efficient and drought-tolerant tomato varieties.

### Shoot morphology and growth patterns: adaptive strategies for water conservation

The observed differences in whole plant leaf area and growth patterns between LEA and Lost highlight distinct adaptive strategies. Lost consistently exhibits a smaller leaf area, aligning with well-established drought adaptation mechanisms in plants from arid environments (Fletcher *et al.*, 2018; Yavas *et al.*, 2024), minimizing water loss through transpiration, and contributing to higher water-use efficiency. Interestingly, Lost's growth pattern, where it overtakes LEA in height around 29 d after transplanting, suggests a nuanced adaptive strategy that balances water conservation and light growth pattern bears similarities to the ‘pessimistic’ growth strategy observed in some desert plants, where growth is initially conservative but can rapidly accelerate vertical growth to enhance light capture with a deeper extensive root system for water uptake (Gutterman, 2012; Feldman *et al.*, 2018; Cui *et al.*, 2019; Wu *et al.*, 2022). Conversely, LEA's consistently higher whole plant-leaf area, associated with greater transpiration and reduced water use efficiency, may explain its lower drought tolerance. Larger leaf area also indicates a strategy to optimize light capture in favourable environments. This is likely an adaptation to maximize photosynthetic efficiency and biomass production when water availability is not limiting (Kégbé *et al.*, 2025), as is often the case in commercial environments.

### Enhanced photosynthetic efficiency

The higher photosynthetic capacity and enhanced net CO_2_ fixation rates per unit leaf area in Lost compared with LEA, reflected by the higher maximum rate of carboxylation (*V*_c,max_) (Fig. 2), can be attributed to both a higher Rubisco content and greater initial activity (Supplementary Fig. S5). These findings provide a biochemical basis for Lost’s superior photosynthetic capacity per unit leaf area, indicating an investment in enhancing the Calvin cycle capacity, often a limiting factor in C_3_ photosynthesis (Parry *et al.*, 2013). The findings here suggest that superior photosynthetic capacity per unit leaf area in Lost is due to a greater investment into Rubisco and other Calvin cycle enzymes (demonstrated by general increase in TSP/total chlorophyll). Several studies have shown that the capacity of CO_2_ assimilation by Rubisco in the leaves of crop species can be improved by increasing the abundance of the specific enzyme in the leaves (see Croce *et al.*, 2024; Salesse-Smith *et al.*, 2025). However, such high investment in Rubisco represents a substantial nitrogen cost per unit leaf area. This suggests that there is likely an optimal level of Rubisco investment, which is finely tuned to environmental conditions (Evans and Poorter, 2001; Flexas and Medrano, 2002; Long *et al.*, 2004).

Higher maximum electron transport rate (*J*_max_) in Lost suggests an enhanced capacity for regenerating RuBP (Supplementary Fig. S5) supporting greater Calvin cycle capacity. The greater *J*_max_ values are reinforced by higher quantum yields (Φ_PSII_ and YI) as well as ETRII and ETRI (Fig. 3), demonstrating an optimization of light harvesting capacity in Lost relative to LEA to support the higher demands for ATP and NADPH for the Calvin cycle. Taken together, Lost has optimized light-harvesting and energy conversion processes to support increased downstream photosynthetic capacity. Such optimization can involve adjustments in the stoichiometry of photosystems and light-harvesting complexes, as well as enhanced regulatory mechanisms for energy distribution between the two photosystems (Eberhard *et al.*, 2008). The enhanced electron flow at both PSII and PSI could be attributed to the optimized structural components of the electron transport chain, including higher relative abundances of reaction centres and cytochrome *b*_6_*f* complexes, as suggested by Schöttler and Tóth (2014). A greater relative abundance of PSII reaction centres compared with light harvesting antennae is supported by the higher ratio of chlorophyll *a* and *b* in Lost (Supplementary Fig. S5) (Anderson and Melis, 1983).

The fact that higher PSI donor side limitation (YND) was observed in LEA compared with Lost (Fig. 3) demonstrates slow delivery of electrons from PC and possible PSII limitation (Schansker, 2022). The greater ‘sink’ capacity for electrons in Lost to support carbon fixation and optimization of electron transport is illustrated by higher Φ_PSII_ due to a greater *F*_q_′/*F*_v_′, which measures the ability of PSII to maintain the plastoquinone pool in the oxidized state and the ability of excited PSII reaction centres to drive electron transport, rather than changes in *F*_v_′/*F*_m_′, which provides an indication of non-photochemical quenching (Baker, 2008; Murchie and Lawson, 2013). The higher demand for electrons in Lost is further supported by the lower Fd_red_ percentage compared with LEA, with electrons being used to reduce Fd and NADP^+^ (Schreiber, 2017; Schansker, 2022), which would be required to support the increased Calvin cycle activity observed here. Fd not only catalyses the reduction of NADP^+^ to NADPH via Fd–NADP^+^ reductase (FNR) (Rochaix, 2013), but also reduces thioredoxin (TRX) the key redox regulator of many key enzymes in the Calvin cycle (Buchanan, 1980; Knaff, 1996; Buchanan *et al.*, 2002), including sedoheptulose-1,7-bisphosphatase, which has been shown to have high control over RuBP regeneration (Lawson *et al.*, 2006). The higher Fd_red_ percentage in LEA indicates a reduced metabolic demand for electrons. Taken together, these data demonstrate a well-regulated electron transport chain with high flux in Lost that does not result in the accumulation of reduced intermediates due to high downstream demand (von Caemmerer, 2000). The investment in carbon fixation relative to light harvesting in Lost is demonstrated by the lower chlorophyll:Rubisco ratio. No differences in the relationship between P700_red_ and *F*_q_′/*F*_v_′ (*q*_P_) were observed between Lost and LEA, with both showing a strong linear relationship (Fig. 4), indicating balanced flow between the two photosystems (Baker *et al.*, 2007; Degen and Johnson, 2024), which is important to prevent photodamage to PSI (Terashima *et al.*, 1994), and no photosynthetic control on electron flow or the accumulation of reduced intermediates.

*A* and *J*_max_ were ca. 2.5 times higher in Lost than LEA (Fig. 2), whilst electron transport rate (ETRI and ETRII) (Fig. 3) was only 20–30% greater. These differences can be attributed to several factors. First, as tomato is a C_3_ plant, the relationship between ETR and *A*, although correlated, is not always linear due to alternative electron sinks including photorespiration or cyclic electron flow. Second, *J*_max_ and *A* from *A–C*_i_ curves represent a maximum capacity (with no limitation from [CO_2_] and/or light intensity), whilst maximum ETR is determined at maximum light intensity only.

The superior electron sink capacity and more efficient electron flow observed in Lost are consistent with its adaptation to arid, high-light environments typical of wild *S. pennellii* habitats (Zhou *et al.*, 2018; Torgeman *et al.*, 2024). In such conditions, efficient use of light energy and rapid electron transport are critical to maximize carbon fixation and minimize photodamage. In contrast, the greater PSI donor side limitation and slower electron delivery in LEA reflect a strategy suited to more stable, resource-rich agricultural environments, where greater leaf area compensates for reduced photosynthetic capacity but requires resource inputs. Furthermore, the higher leaf area in LEA most likely results in a greater proportion of leaf area under self-shading, and therefore a higher capacity in photosynthesis is not required, and investment in light harvesting is of greater importance.

While Lost exhibits superior photosynthetic capacity per unit leaf area (e.g. higher *A*, *V*_c,max_, *J*_max_), it is important to acknowledge that the smaller plant size and lower total leaf area per plant means that capacity for light harvesting is compromised at the whole plant level. As a result, photosynthetic rate per plant may be lower than LEA.

These physiological differences likely underpin each species’ adaptation to its native environment, with Lost optimized for fluctuating, stressful conditions and LEA for managed cultivation. Lost’s efficient electron transport and greater photosynthetic capacity, supported by thicker leaves and higher protein content per unit leaf area, enable robust carbon fixation even with smaller, more compact leaves and lower SD. These traits can be beneficial in agriculture by improving productivity and stress tolerance under high light and drought, while reducing water loss—making Lost a valuable source for breeding more resilient, resource-efficient tomato crops in specific environments. The fact that Lost, a wild relative, has these beneficial traits suggests that they may have disappeared through domestication and selective breeding for other key agronomic traits.

### Stomatal characteristics: balancing gas exchange and water conservation

In most dicotyledonous plants, a defining feature of leaf anatomy is the distinct asymmetry in stomatal distribution. The abaxial surface generally has a much higher SD compared with the adaxial surface (Pemadasa, 1979; Mott *et al.*, 1982; Willmer and Fricker, 1996), thought to enhance water conservation from high evaporative demand (Willmer and Fricker, 1996) while optimizing gas exchange efficiency (Fanourakis *et al.*, 2015; Muir, 2019). The equal SD on both leaf surfaces in Lost (Fig. 5A), contrasting with the typical dicotyledonous pattern of higher abaxial density in LEA, underlines a significant physiological adaptation. The contrasting patterns of *g*_s_ and *A* observed between LEA and Lost (Fig. 6) provide valuable insights into the distinct photosynthetic strategies employed by these tomato species. The higher *g*_s_ on the abaxial leaf surface of LEA and lower *g*_s_ on the adaxial surface are consistent with SD patterns (as there were no differences in pore size; Fig. 5B), and aligns with the established understanding of stomatal control on gaseous fluxes, in which low distribution on the adaxial surface is associated with reducing water loss, where evaporative demand maybe high due to solar radiation (Muir, 2019). The uniform amphistomatous SD pattern and *g*_s_ in Lost hints at alternative regulatory mechanisms, in which stomata operate independently on the two surfaces, and is often found in plants from high-light, water-limited environments (Drake *et al.*, 2019), although the resulting functional differences on overall leaf gas exchange have been less well studied (Mott *et al.*, 1982; Xiong and Flexas, 2020; Santos *et al.*, 2021; Wall *et al.*, 2022). Previous studies have suggested that amphistomaty is associated with a superior capacity for gaseous diffusion through a reduced diffusion pathlength for CO_2_ (Parkhurst, 1978, 1994; Franks and Beerling, 2009), supporting higher photosynthesis rates (Richardson *et al.*, 2020), and gas exchange from both surfaces is essential to meet the demands of a thicker leaf. The advantage of this arrangement lies in maximizing CO_2_ uptake per unit of water lost, as it allows for a more even distribution of gas exchange across the leaf surface (Drake *et al.*, 2019; Xiong and Flexas, 2020) and has been reported to be advantageous for plants adapted to high light environments (Muir, 2019)—such as Lost. The significant decoupling of abaxial *A* and *g*_s_ in Lost indicates that this surface contributes significantly to CO_2_ uptake, as well as achieving high water use efficiency. Similar patterns of one surface dominating gas exchange have been observed in wheat (Wall *et al.*, 2022), although the adaxial surface was the greatest contributor in this species. The higher assimilation rate on the abaxial side despite similar *g*_s_ to the adaxial side suggests that Lost possesses a mechanism(s) to enhance CO_2_ assimilation on the abaxial side, independent of stomatal control. This could be due to enhanced mesophyll conductance owing to denser spongy mesophyll (Supplementary Fig. S4), facilitating CO_2_ diffusion to the chloroplasts and/or enhanced photosynthetic capacity in the mesophyll cells adjacent to this surface (Wall *et al.*, 2022; Fan *et al.*, 2025). Alternatively, this could suggest that *g*_s_ on the adaxial surface is greater than required to support maximum *A*, which is supported by the fact that LEA also maintains high *g*_s_ for the same rates of *A* (Fig. 5).

Previous studies have reported that *S. pennellii* exhibits similar *g*_s_ to *S. lycopersicum* under normal conditions (Egea *et al.*, 2018; Dariva *et al.*, 2020; Lana-Costa *et al.*, 2020). However, our findings reveal that LEA has a higher *g*_s_ than Lost. It is important to acknowledge that natural and intraspecific variation within *S. pennellii* can lead to differences in stomatal traits and physiological responses across accessions and environments. These variations, besides unequal SD, may account for the discrepancies in *g*_s_ observed in wild and cultivated tomato in our results with previous reports.

### Leaf anatomy

The observation that leaves of Lost are thicker than those of LEA is crucial for understanding its superior photosynthetic performance. Thicker leaves are often linked to higher photosynthetic rates per unit leaf area, due to increased chloroplast density (Pauli *et al.*, 2017), evidenced here by the increased mesophyll cell density and the higher Rubisco activity in Lost (Supplementary Fig. S4) to support greater photosynthetic capacity. The higher mesophyll cell density provides a larger surface area for CO_2_ diffusion as well as more chloroplasts for carbon fixation, resulting in higher mesophyll conductance (Flexas *et al.*, 2008; Huang *et al.*, 2022) (Supplementary Fig. S4). These anatomical adaptations may partially explain how Lost achieves higher *A* with lower *g*_s_ and aligns with the observed higher Rubisco activity and content, indicating a more rapid Calvin cycle turnover and greater CO_2_ fixation (Parry *et al.*, 2013). For Lost to have a higher Rubisco amount per unit leaf area to support photosynthesis, nitrogen concentration per area increases, and to achieve this, a thicker leaf is required, increasing LMA. As thicker leaves cost more biomass to be produced, thinner leaves represent a classic resource allocation trade-off. For Lost to have an equivalent light harvesting capacity at the whole plant level to LEA, if needed, it would need higher resource (e.g. N) investment per unit leaf area. Leaf thickness has previously been recognized as a key determinant of photosynthetic efficiency and yield in other crops including rice and cotton (Pauli *et al.*, 2017; Huang *et al.*, 2022).

The uniform stomatal distribution (similar SD on both leaf surfaces) observed in Lost may work synergistically with this dense mesophyll arrangement to enhance gas exchange efficiency across the entire leaf, particularly on the abaxial surface. While we did not quantify specific anatomical parameters from the microtomy sections, the qualitative differences in mesophyll structure suggest that the combination of uniform stomatal distribution and dense mesophyll tissue creates favourable conditions for CO_2_ diffusion and utilization, especially at the typically more photosynthetically active abaxial surface.

### Influence of rootstocks on physiology and performance

To determine whether any photosynthetic-related traits in Lost could be transferred to LEA, self and reciprocal grafting between LEA and Lost was performed. The observed patterns of SD in self-grafted plants (Fig. 7), which are consistent with SD from non-grafted plants (Fig. 5), indicates that the grafting process itself does not significantly alter the inherent stomatal distribution patterns. However, the most intriguing findings coming from the analysis of reciprocal grafts were the reduced difference in SD between abaxial and adaxial surfaces in LEA/Lost grafts compared with non-grafted and self-grafted LEA. This partial shift towards an amphistomatous pattern, concurrent with changes in size, indicates that the Lost rootstock exerts some influence on the stomatal development of the LEA scion potentially through hormonal or nutritional signalling pathways. These changes may partially explain the higher photosynthetic assimilation in LEA/Lost compared with non-grafted and self-grafted LEA. However, this is not only a rootstock signal, as Lost/LEA grafts maintained the amphistomatous pattern characteristic of Lost, suggesting that the stomatal distribution in the scion is primarily determined by the genetic makeup of Lost. These observations align with studies on long-distance signalling in grafted plants as well as potential differences in root/stem hydraulic conductance. For example, Albacete *et al.* (2015) demonstrated that rootstocks can influence various physiological processes in the scion, including leaf development and stomatal behaviour, through the transport of hormones, small RNAs, and other signalling molecules. To determine whether the altered SD pattern in grafted plants affected photosynthetic performance, gas exchange measurements were conducted on the various grafting combinations and non-grafted controls (Fig. 8). The successful transfer of enhanced photosynthetic traits from Lost to LEA through grafting is a significant finding with immediate practical implications. This non-GM approach offers a promising avenue for improving drought tolerance, photosynthetic efficiency, and overall crop performance in cultivated tomatoes. The success of this method suggests that traits related to root-to-shoot signalling or resource allocation observed in Lost can be conferred through the rootstock. This approach leverages the robust root system and physiological traits of wild relatives, as demonstrated in other studies that improved quality, yield and stress tolerance of commercial tomato lines (Gao *et al.*, 2017; Zhang *et al.*, 2019; Fuentes-Merlos *et al.*, 2022; Parisi *et al.*, 2023; Bahadur and Kumar, 2024). Future work should examine the long-term stability and heritability of these traits, as well as the photosynthesis-related metabolites transported from rootstock to scion through the xylem and the underlying genes as well as molecular mechanisms enabling rootstock–scion communication. Besides, a comparative analysis of root morphology between LEA and Lost may provide valuable insights.

## Conclusion and future direction

This comprehensive comparative study of LEA and Lost has uncovered a complex array of physiological and anatomical adaptations that underpin the exceptional photosynthetic efficiency of Lost. The wild species sustains high photosynthetic rates through a combination of strategies, including optimized leaf morphology for water conservation, efficient stomatal distribution and regulation, elevated Rubisco content and Calvin cycle activity, balanced with high electron transport capacity. These characteristics are most likely key adaptive strategies in Lost, which is typically native to arid and semi-arid environments of western South America, notably Peru and northern Chile, characterized by high temperature, low rain fall, and challenging growing conditions. These findings not only deepen our understanding of plant adaptations to arid environments, but also offer valuable insights for crop improvement strategies and the discovery of novel traits from wild relatives that may infer crop resilience and greater productivity. Notably, the successful transfer of enhanced photosynthetic traits from Lost to LEA through grafting highlights the potential to incorporate these adaptive traits into cultivated tomato varieties as well as provide a greater understanding of root to shoot signalling and hydraulic architecture.

## Supplementary Material

eraf390_Supplementary_Data

## Data Availability

Data are available at the University of Essex repository (Ganie *et al*., 2025). https://doi.org/10.5526/ERDR-00000226

## Supplementary data

The following supplementary data are available at *JXB* online.

Fig. S1. Redox state of photosynthetic components in LEA and Lost under varying light intensities (PAR).

Fig. S2. Kinetic parameterization of stomatal and photosynthetic responses to a step change in light intensity.

Fig. S3. Kinetic parameterization of stomatal and photosynthetic from both the adaxial and abaxial surface following a step change in light intensity.

Fig. S4. Leaf thickness and leaf mass per area (LMA) in LEA and Lost.

Fig. S5. Comparison of biochemical and photosynthetic traits in LEA and Lost.

Fig. S6. Photosynthetic capacity traits across grafting combinations between LEA and Lost genotypes.
