# Supplementary material for: Unravelling the physiological and anatomical basis of divergent adaptations in cultivated and wild tomatoes
Source: J Exp Bot. 2025 Sep 4;76(21):6548–66. doi: 10.1093/jxb/eraf390 (PMC12646152; doi:10.1093/jxb/eraf390)
Supplement: eraf390_Supplementary_Data [file eraf390_supplementary_data.pdf]

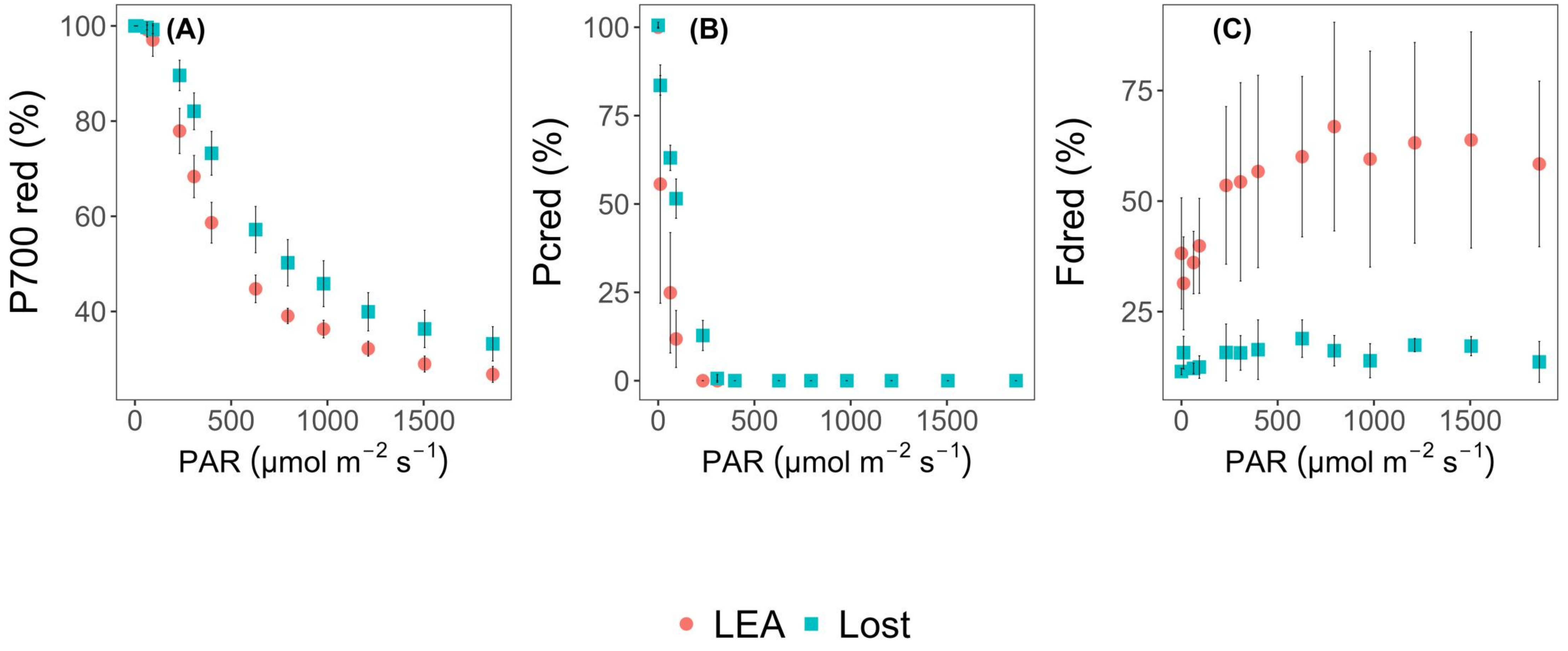

Supplementary Fig. S1. Redox state of photosynthetic components in LEA and Lost under varying light intensities (PAR). (**A**) Percentage of reduced P700 (P700 red (%)) decreases with increasing PAR in both genotypes. LEA exhibits a more pronounced decrease compared to Lost. (**B**) Percentage of reduced plastocyanin (Pcred (%)) decreases with increasing PAR and remains near zero at high PAR in both genotypes. (**C**) Percentage of reduced ferredoxin (Fdred (%)) remains somewhat constant at low values for Lost. The Fdred (%) increases initially with PAR for LEA but begins to decline at high PAR. Data are presented as mean values ± standard deviation (n = 3-6).

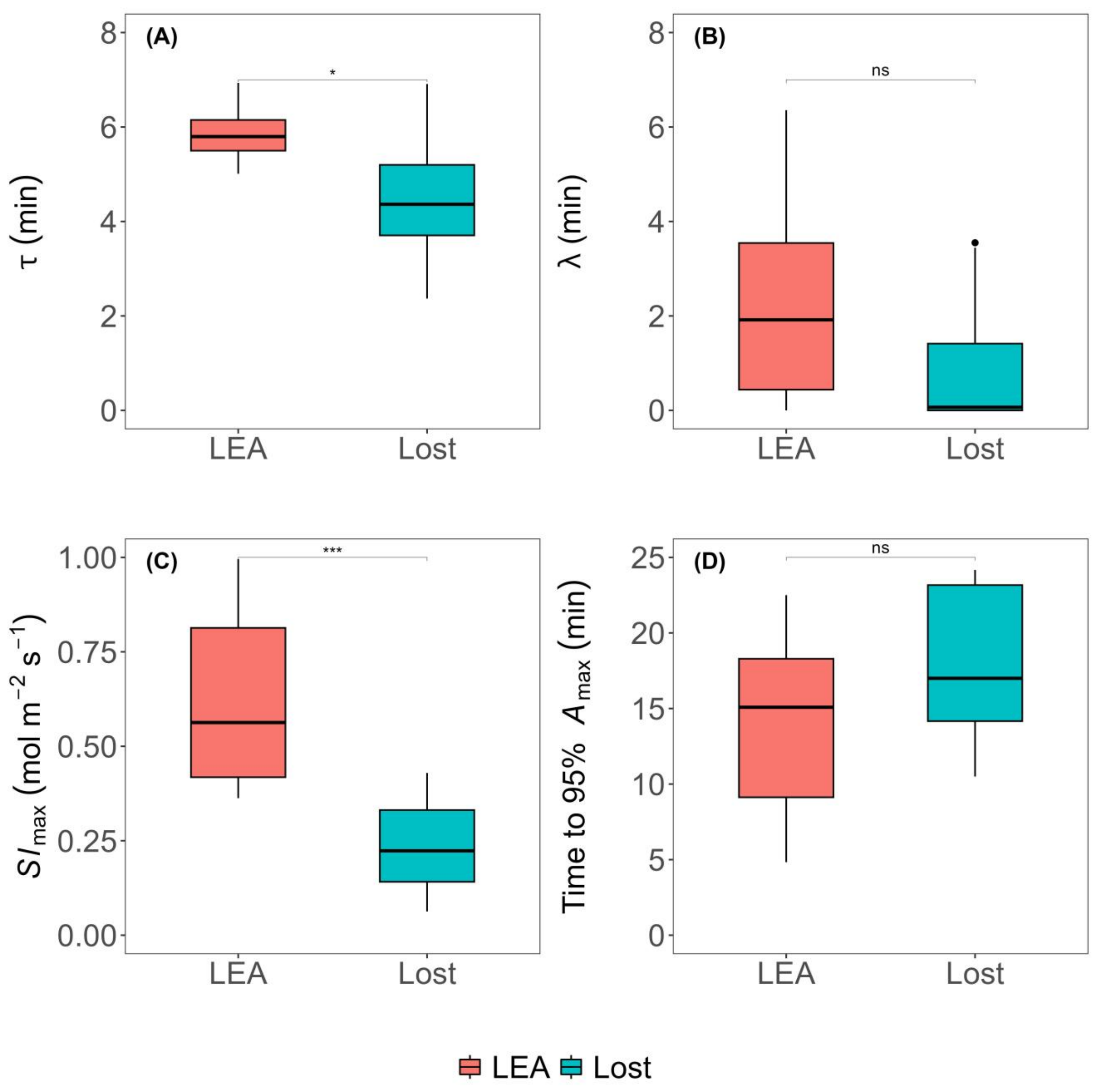

Supplementary Fig. S2: Kinetic parameterization of stomatal and photosynthetic responses to a step change in light intensity. (**A**) The time for g_s to reach 63% of the exponential response (τ); (**B**) Lag time (λ) before stomatal opening; (**C**) Maximum slope of stomatal opening (Sl_{max}); (**D**) Time to reach 95% of maximum assimilation (A_{max}). Data are presented as mean values ± standard deviation (n = 3-6). Statistical differences were assessed using a Student's t-test except when asumptions are not validated where Mann-Whitney test was used. Asterisks indicate significance (*p<0.05, ***p<0.001, ns = not significant).

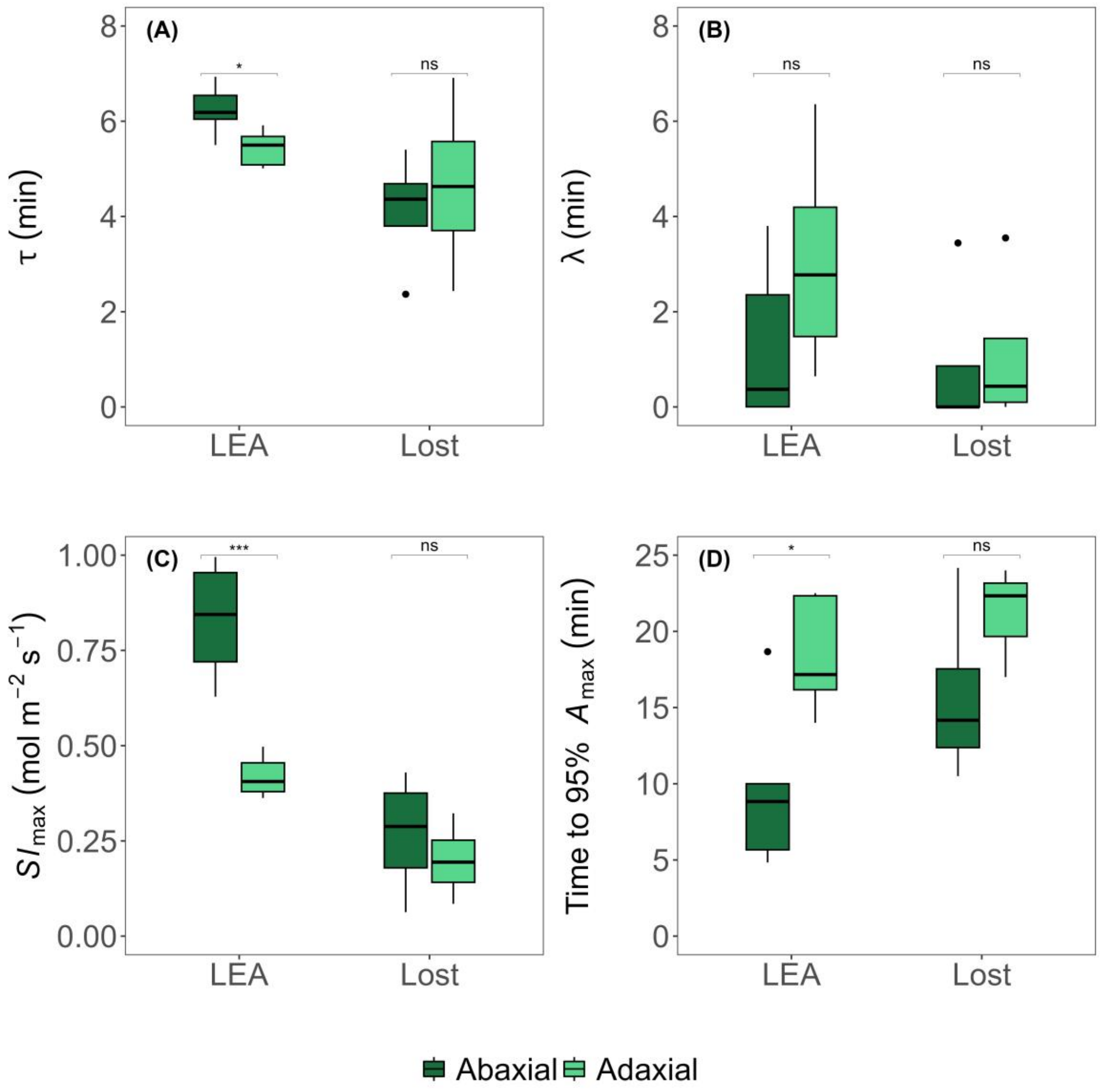

Supplementary Fig. S3: Kinetic parameterization of stomatal and photosynthetic from both the adaxial and abaxial surface following a step change in light intensity. (**A**) The time for g_s to reach 63% of the exponential response (τ); (**B**) Lag time (λ) before stomatal opening; (**C**) Maximum slope of stomatal opening (Sl_{max}); (**D**) Time to reach 95% of maximum assimilation (A_{max}). Data are presented as mean values ± standard deviation (n = 3-6). Statistical differences were assessed using a Student's t-test except when assumptions are not validated where Mann-Whitney test was used. Asterisks indicate significance (*p<0.05, ***p<0.001, ns = not significant).

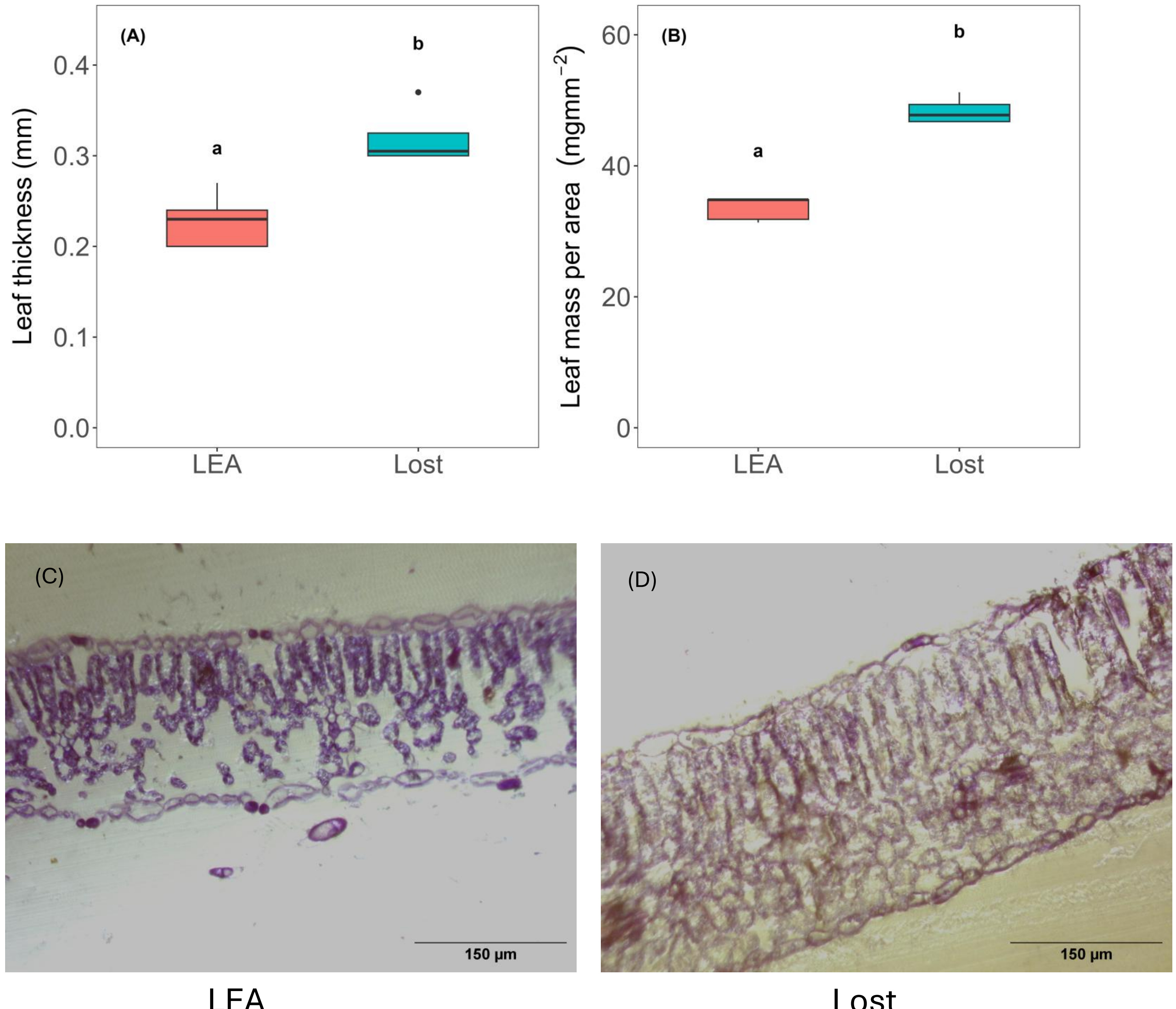

Supplementary Fig. S4. Leaf thickness and leaf mass per area (LMA) in LEA and Lost. Lost exhibits significantly greater (**A**) leaf thickness ($p<0.05$) and (**B**) LMA ($p<0.05$) than LEA. (**C-D**) Mesophyll structure and arrangement in LEA and Lost. Lost exhibited larger, more densely packed palisade mesophyll cells and higher spongy mesophyll cell density than LEA. Data are presented as mean values ± standard deviation (n = 3-6). Statistical differences were assessed using a Mann-Whitney test. Different letters denote significant differences between genotypes ($p<0.05$).

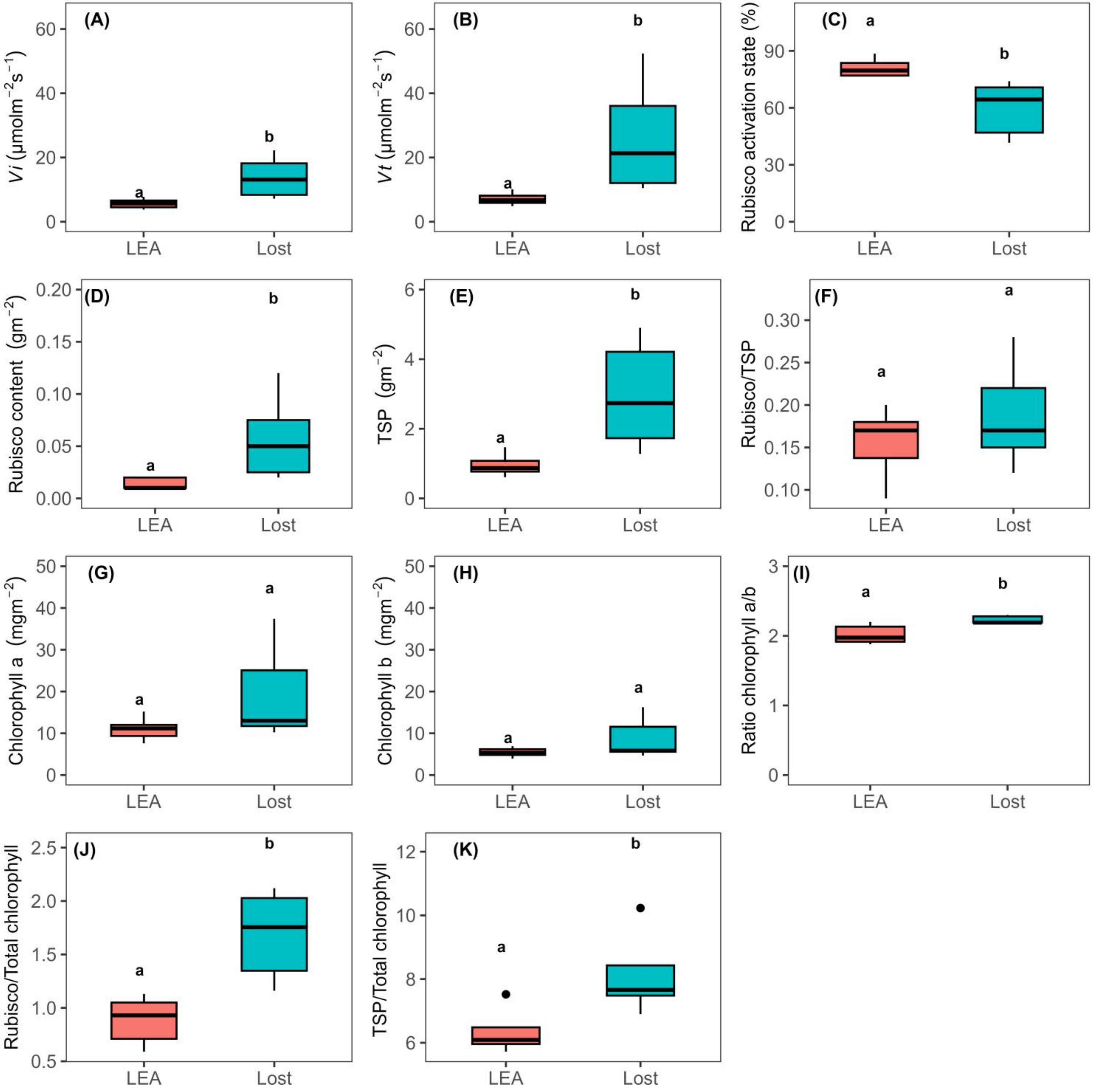

Supplementary Fig. S5 Comparison of biochemical and photosynthetic traits in LEA and Lost. (**A-C**) Lost exhibits significantly higher initial (V_i) and total (V_t) Rubisco activity compared to LEA (p<0.05, p<0.05, respectively). (**D-F**) Rubisco and total soluble protein content (TSP) contents are also considerably greater in Lost (p<0.05, respectively), correlating with its higher photosynthetic assimilation. (**G-I**) Chlorophyll content was not significantly different between the two species; however, the ratio of chlorophyll a and chlorophyll b was significantly higher in Lost (p<0.05) (**J-K**) Additionally, Ratio of Rubisco and TSP to total chlorophyll content is significantly higher in Lost than LEA (p<0.01, p<0.05, respectively). Data are presented as mean values ± standard deviation (n = 3-6). Statistical differences were assessed using a Student's t-test for (C), (F), (I), (J), (K); Welsh's test for (A), (B), (E); and Man-Whitney's test for (D), (G), (H). Different letters denote significant differences between genotypes (p<0.05).

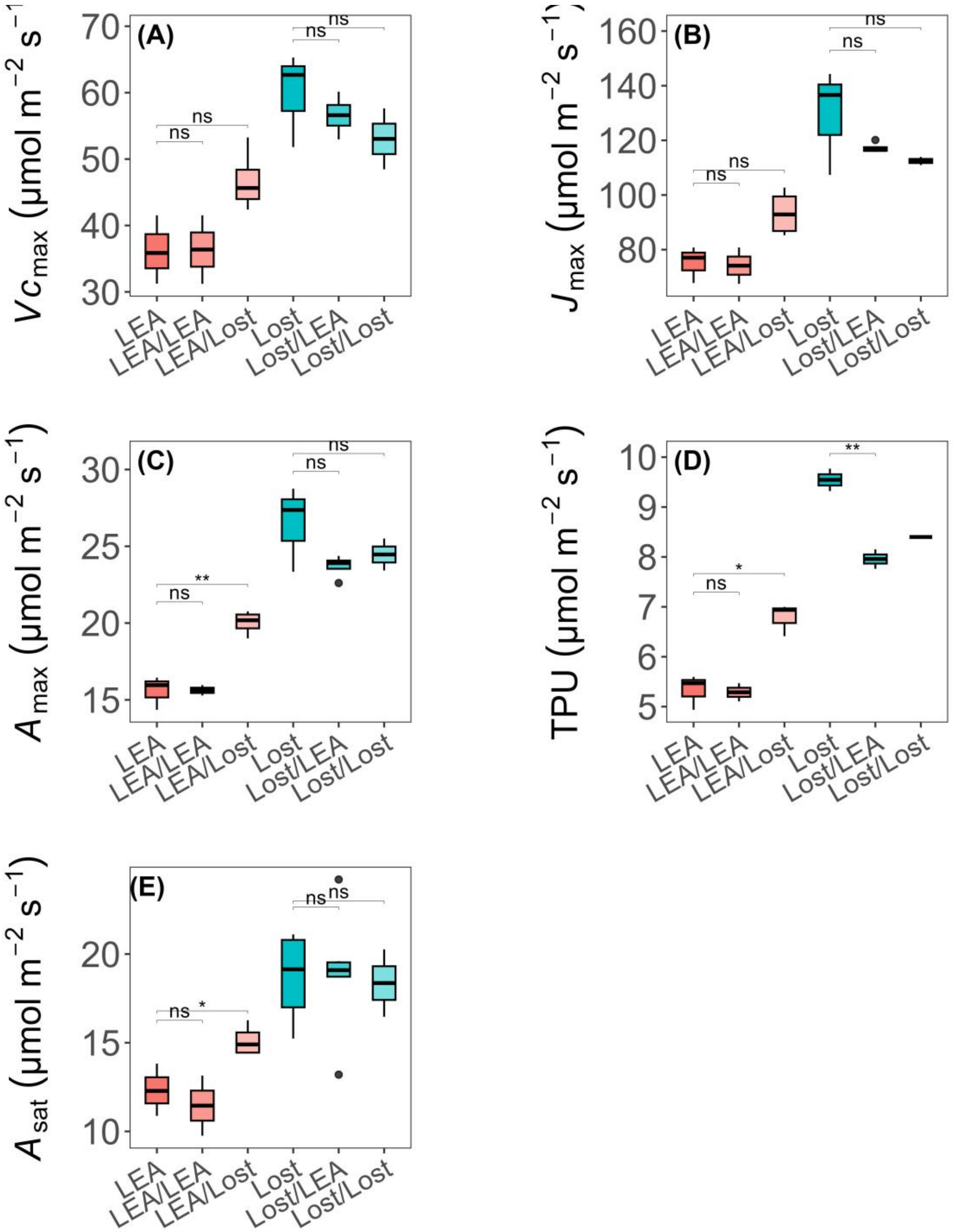

Supplementary Fig. S6. Photosynthetic capacity traits across grafting combinations between LEA and Lost genotypes. Photosynthetic parameters derived from AC*i* curves – including (**A-D**) maximum Rubisco carboxylation rate (Vc_{max}), maximum electron transport rate (J_{max}), maximum assimilation rate (A_{max}), and triose phosphate utilization (TPU) – and AQ curves – including (**E**) light-saturated assimilation (A_{sat}) are shown for non-grafted controls (LEA, Lost) and four grafted combinations: LEA/Lost, Lost/LEA, LEA/LEA, and Lost/Lost. Comparisons are made relative to their respective non-grafted controls. LEA/Lost grafts (LEA scions on Lost rootstocks) show significantly higher A_{max}, TPU, and A_{sat} than non-grafted LEA, suggesting a strong influence of Lost rootstocks in enhancing photosynthetic performance of LEA scions. In contrast, grafting Lost onto LEA (Lost/LEA) results in limited improvement over non-grafted Lost. Data are presented as mean values ± standard deviation (n = 3-6). Statistical differences were assessed using a Student's t-test. Asterisks indicate significance (*** p<0.001; ** p<0.01; * p<0.05; ns = not significant).
